# Role of the human vaginal microbiota in the regulation of inflammation and sexually transmitted infection acquisition: Contribution of the non-human primate model to a better understanding?

**DOI:** 10.3389/frph.2022.992176

**Published:** 2022-12-06

**Authors:** Cindy Adapen, Louis Réot, Elisabeth Menu

**Affiliations:** ^1^ Micalis Institute, AgroParisTech, INRAE, Université Paris-Saclay, Jouy-en-Josas, France; ^2^ Université Paris-Saclay, Inserm, Commissariat à l'énergie Atomique et aux énergies Alternatives (CEA), Center for Immunology of Viral, Auto-Immune, Hematological and Bacterial Diseases (IMVA-HB)/Department of Infectious Disease Models and Innovative Therapies (IDMIT), Fontenay-aux-Roses, France; ^3^ Mucosal Immunity and Sexually Transmitted Infection Control (MISTIC) Group, Department of Virology, Institut Pasteur, Paris, France

**Keywords:** vaginal microbiota, inflammation, cytokines, neutrophils, sexually transmitted infections, non-human primates

## Abstract

The human vaginal microbiota has a central role in the regulation of the female reproductive tract (FRT) inflammation. Indeed, on one hand an optimal environment leading to a protection against sexually transmitted infections (STI) is associated with a high proportion of *Lactobacillus* spp. (eubiosis). On the other hand, a more diverse microbiota with a high amount of non-*Lactobacillus* spp. (dysbiosis) is linked to a higher local inflammation and an increased STI susceptibility. The composition of the vaginal microbiota is influenced by numerous factors that may lead to a dysbiotic environment. In this review, we first discuss how the vaginal microbiota composition affects the local inflammation with a focus on the cytokine profiles, the immune cell recruitment/phenotype and a large part devoted on the interactions between the vaginal microbiota and the neutrophils. Secondly, we analyze the interplay between STI and the vaginal microbiota and describe several mechanisms of action of the vaginal microbiota. Finally, the input of the NHP model in research focusing on the FRT health including vaginal microbiota or STI acquisition/control and treatment is discussed.

## 1. Introduction

The female reproductive tract (FRT) is one of the entry points of sexually transmitted pathogens and includes a wide array of immune parameters necessary to abrogate pathogen invasion. The FRT is divided into an upper tract, composed of the Fallopian tubes, ovaries, uterus and endocervix and a lower tract, with the ectocervix and the vagina. The two compartments differ in their architecture, with two distinct types of mucosae (type I and II) (1, 2) The critical zone for pathogen invasion is the transformation zone, located between the upper and lower tract, where the multilayer squamous epithelium changes to a single layer of columnar epithelial cells (3). The transformation zone is immunologically active, the change of epithelium architecture allows the passage of small pathogens to the mucosa. The lower tract epithelium permits the protection of underlining tissues from abrasion during intercourse. However, the lack of tight junction allows intra-epithelial passage of molecules, small pathogens and interaction of these pathogens with potential target cells. The epithelial barrier in the upper and lower tracts acts as a physical barrier but can also recognize Pathogen Associated Molecular Patterns (PAMPs) through Pattern recognition receptors (PRR) like Toll-like receptors (TLR) and NOD-like receptors (NLR) and induces the production of cytokines, chemokines and antimicrobial peptides (AMP) (4, 5). A layer of mucus, covering the epithelium of the FRT, is in direct contact with pathogens. The mucus acts as a physical trap for pathogens thanks to gel-like properties (6).

Macrophages, NK cells, dendritic cells (DC) and neutrophils have a variable distribution according to FRT compartments and hormonal levels (4, 7). Other innate-like lymphocyte cells such as γδ and mucosal-associated invariant T (MAIT) cells are also present within the FRT (8, 9). T lymphocytes are the most abundant leukocytes in the FRT (10, 11). The transformation zone contains the highest concentration of macrophages, CD4+ and CD8+ T cells compared to all FRT compartments (12). IgA and IgG are found in the FRT, and IgG concentration is higher than IgA in cervicovaginal fluids (13). B cells are mainly found in the cervix and vagina and are more abundant in the ectocervix compared to the endocervix in premenopausal women (11, 14).

Hormonal changes in the FRT induce several modifications of the immune system involving secreted molecules (AMPs, cytokines and chemokines), epithelial cells, fibroblasts, immune cells and vaginal microbiota (14).

**Figure 1:**
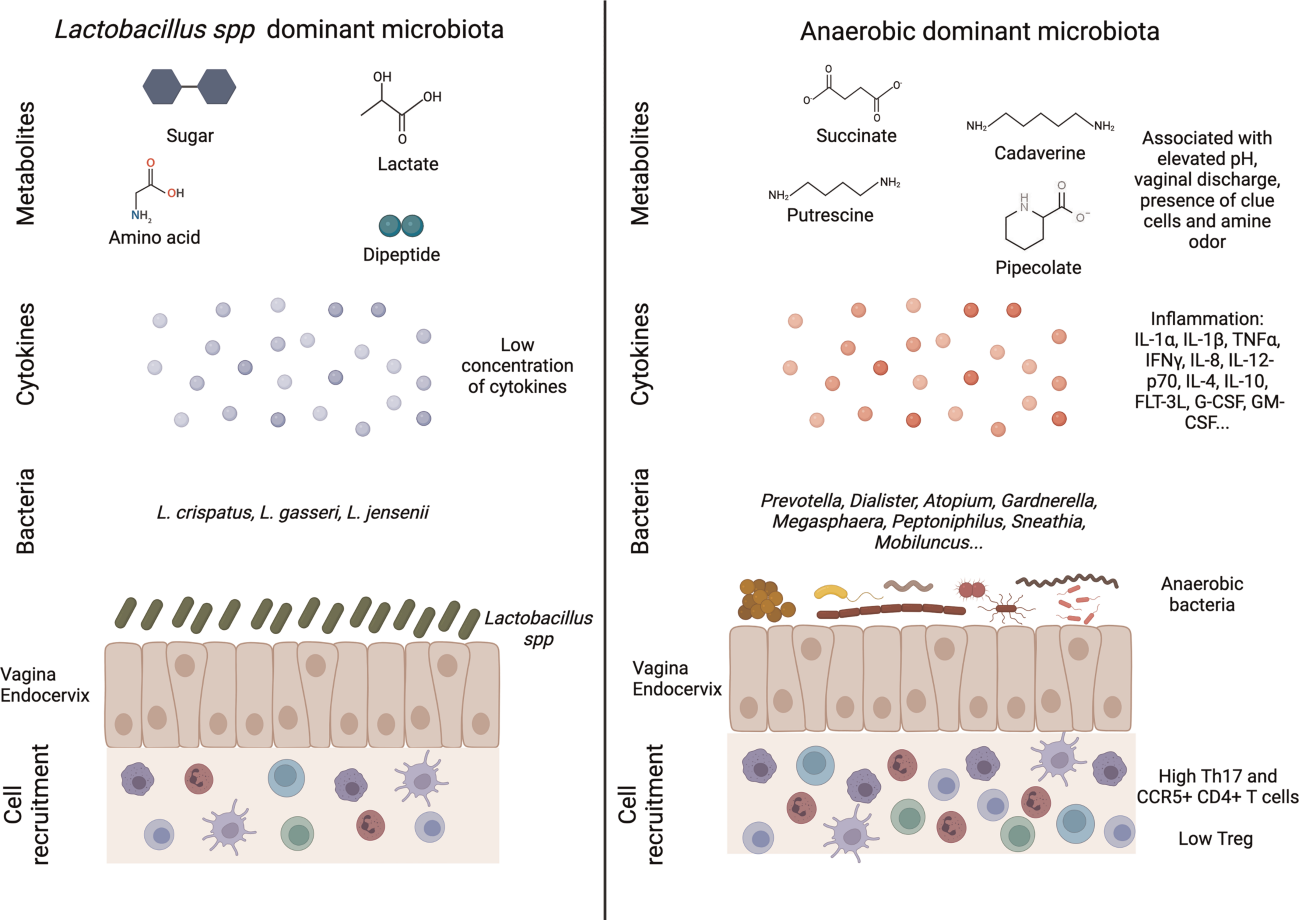
Vaginal microbiota and female reproductive tract inflammation. An eubiotic vaginal microbiota is dominated by *Lactobacillus* spp. that can produce different metabolites such as sugar, lactate, amino acid and dipeptide. This environment is associated with a low inflammation including cytokine production and immune cells recruitment (**left panel**). On the contrary, a more diverse vaginal microbiota (low *Lactobacillus* spp. and high anaerobic bacteria abundances) is associated with an increased inflammation. The microbial environment leads to the production of metabolites linked to vaginal discomfort such as vaginal discharges or amine odor. In this context, higher production of cytokines and recruitment of T cells (Th17 and CCR5+ CD4+ T cells) are detected (**right panel**). Created with BioRender.com.

In humans, eubiotic vaginal microbiota is characterized by the dominance of one genus, *Lactobacillus* spp., which maintains an acidic pH in the vagina (pH = 4–5) thanks to lactic acid production. It also maintains a low inflammatory environment through the production of bactericide and bacteriostatic components (Figure 1). Analysis of the vaginal microbiota by 16S rRNA gene sequencing and vaginal pH measurement allowed a community clustering into five groups or community state type (CST) (15). Group I, II, III and V are dominated by one species of *Lactobacillus* spp.: *L. crispatus* (group I), *L. gasseri* (group II), *L. iners* (group III) and *L. jensenii* (group V). Women from the group IV had a vaginal microbiota characterized by higher proportions of anaerobic bacteria (facultative or strict other than *Lactobacillus* spp.) including *Prevotella*, *Dialister*, *Atopobium*, *Gardnerella*, *Megasphaera*, *Peptoniphilus*, *Sneathia*, *Mobiluncus*, with a vaginal pH starting at 5 and rising up to more than 5,5. The group IV can be divided into two subgroups: characterized by a microbiota with a modest proportion of *Lactobacillus* spp. and a low number of various species of anaerobic bacteria (such as *Anaerococcus*, *Corynebacterium*, *Finegoldia* or *Streptococcus*) (group IVa), or dominated by diverse anaerobic bacteria belonging to the genus *Atopobium*, *Prevotella*, *Parvimonas*, *Sneathia*, *Gardnerella* or *Mobiluncus* (group IVb) (16). This diverse vaginal microbiota composition has been associated with local inflammation, characterized by an increase in pro-inflammatory cytokines and the presence of activated cells, which in turn increase the susceptibility to sexually transmitted infections (STIs) and poor obstetrics events (17, 18) (Figure 1). This increased bacterial diversity associated with high anaerobic bacteria abundance, low *Lactobacillus* spp. abundance and high local inflammation, leads to a clinical condition called bacterial vaginosis (BV). BV is also associated with a modification of metabolomic profiles. In BV+ women, several metabolites are associated with BV clinical criteria: elevated pH (increase of tyramine, cadaverine, N-acetylputrescine, sphingosine and decrease in tyrosine and glutamate), vaginal discharge (increase of cadaverine and agmatine), presence of clue cells (increase of deoxycamitine, pipecolate and decrease of glutathione, glycylproline) and amine odor (increase of N-acetylputrescine and decrease in lactate, phenylalanine). On the contrary, *L. crispatus* and *L. jensenii* abundance have been described to correlate positively with other metabolites such as sugars, lactate, amino acids and dipeptides, but negatively correlated with tyramine, pipecolate, cadaverine putrescine and agmatine, which are increased in BV+ women (Figure 1). *L. iners* exhibited intermediate correlation patterns between *L. crispatus*/*L. jensenii* and BV associated bacteria (19). *Gardnerella vaginalis* is often associated with BV and the predominant agent inducing biofilm. This bacterium was present in all the patients suffering from BV. However, it was also observed in 59% of BV− patients, suggesting that *G. vaginalis* is also present in healthy women (20). *G. vaginalis* alone does not systematically induce BV (21), but has a central role in BV acquisition by being a part of a group of organisms including *Atopobium*, *Megashaera*, *Sneathia* and BVAB1/2/3 involved in the induction of a dysbiotic environment (22).

**Figure 2:**
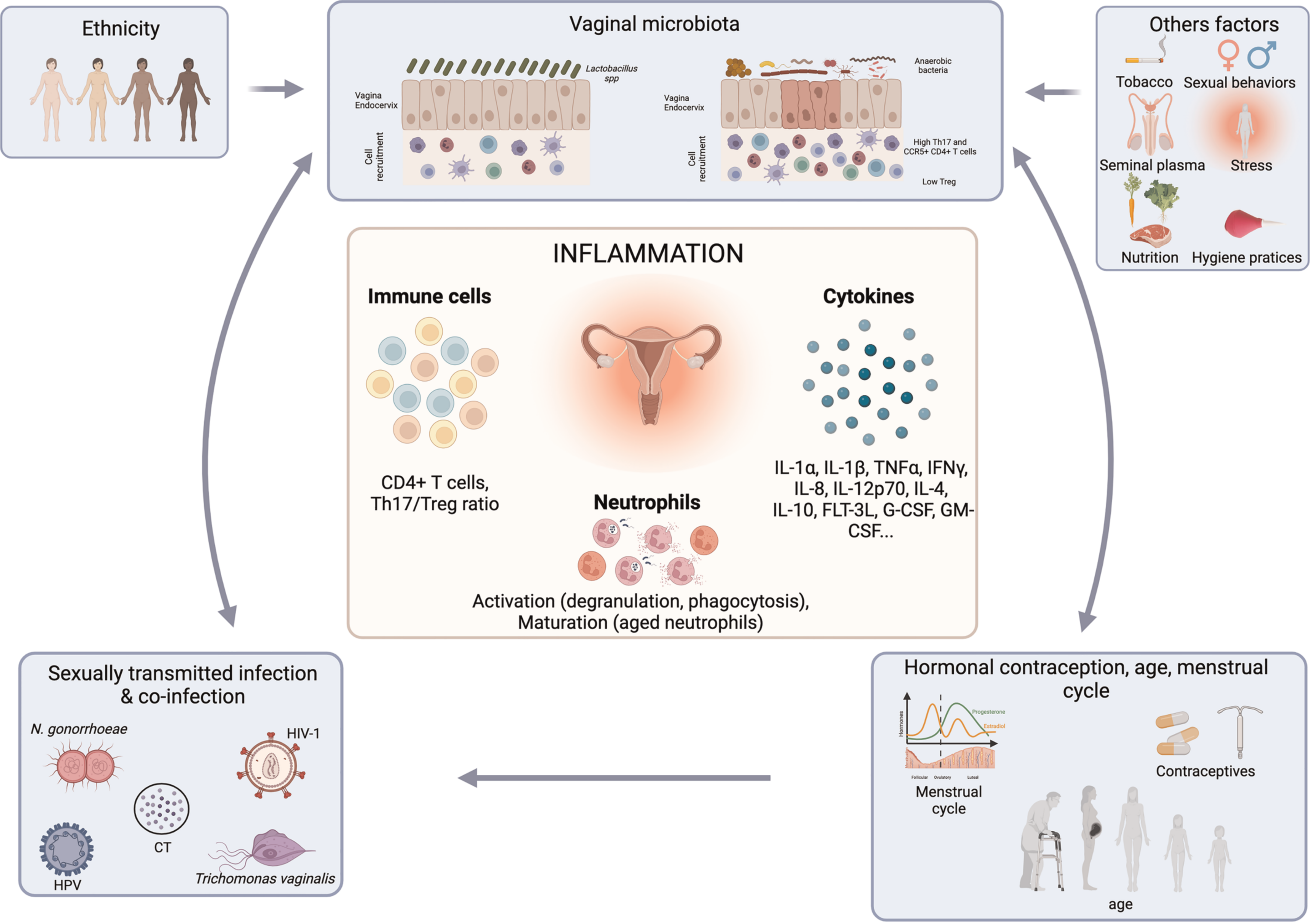
Association between factors affecting the vaginal microbiota and the effect on inflammation. The vaginal microbiota can be influenced by numerous factors including sexually transmitted infection, hormonal contraception, menstrual cycle, ethnicity, sexual behaviors, hygiene practices, stress, nutrition etc. Those factors by influencing the vaginal microbiota will affect the FRT inflammation including cytokine production, T cell recruitment and neutrophil activation and maturation. Created with BioRender.com.

Various factors are known to influence the vaginal microbiota composition, such as hormones, contraceptive use, hygiene, sexual behaviors, semen, ethnic status and STIs (23–32) Other factors including nutrition, stress or tobacco use have also been shown to influence the vaginal microbiota composition (33–36) (Figure 2).

Here, we address the interplay between vaginal microbiota composition and vaginal inflammation at steady state and in the context of a STI. Finally, we discuss animal models and more precisely non-human primate (NHP) use to study vaginal microbiota composition and its link to local inflammation.

## 2. The vaginal microbiota and inflammation

### 2.1. Cytokine production

The vaginal microbiota has been described to be closely linked to FRT inflammation (Figure 1). Women with CST-IV microbiota were observed to be four times more likely to have elevated genital pro-inflammatory cytokines, compared to those with CST-I-III. More precisely, IL-1α, IL-1β, TNFα, IFNγ, IL-8, IL-12-p70, IL-4, IL-10, and FMS-like tyrosine 3 ligand (FLT-3L) were significantly increased in women with CST-IV compared to women with CST-I (17). Moreover, several bacteria were associated with inflammation: *Fusobacterium*, *Aerococcus*, *Sneathia*, *Gemella*, *Mobiluncus* and *Prevotella* (17). BVAB1/2/3, *Prevotella amnii* and *pallens*, *Parvimonas micra*, *Megasphaera*, *G. vaginalis* and *Atopobium vaginae* (37). Elevated concentration of IL-1α, IL-1β and decrease of CXCL10 were proposed to be used as biomarker for asymptomatic STI or BV (38). Jespers et al. associated a better vaginal health with a low concentration of IL-1β, IL-8 and IL-12(p70) but they observed an increase of CXCL10 concentration (39). In another study conducted on cervicovaginal lavages (CVL), obtained from an African cohort, the authors observed an increase of IL-1α, IL-1β, G-CSF, GM-CSF and a decrease of CXCL10 in women suffering from dysbiosis (40). In *in vitro* experiments, an increased inflammation (IL-1β, Il-6, IL-8, G-CSF, PDGF, CXCL10, CCL4 and Gro-α) was detected when cell lines from the FRT were exposed to *G. vaginalis* or *A. vaginae* compared to *L. johnsonii*. However, a difference in CXCL10 expression has to be noted between *in vitro* and *in vivo* analysis (41). Similar results were observed for *A. vaginae* or *G. vaginalis* in a 3D model of human cervix: polymicrobial exposition led to an increase production of IL-1α, IL-6, IL-8, CCL2, CCL20 and IL-1β compared to no bacterial exposition or *L. crispatus* exposition (42).

An analysis of the microbiota and proteome from CVL of Rwandan female sex workers showed that several parameters were increased from groups 1 (*L. crispatus* dominated) to 4 (severe dysbiosis) including histones (HIST3H2A, HIST2H3A/C/D, HIST4H4), AMP (psoriasin, calprotectin), proteasome core complex proteins involved in catabolism and IL-36. MUC5B was also increased from groups 1 (*L. crispatus* dominated) to 4 (severe dysbiosis) and MUC5AC was increased in group 4 compared to other groups, which could be explained by an increased production of mucus, or by the degradation of the mucus by BV associated bacteria. Indeed, *G. vaginalis* was observed to degrade MUC1 compared to *L. crispatus* in a 3D model of human cervix (42). Cytoskeleton alterations (increased actin-organizing proteins, decreased keratins and cornified proteins) were also observed in groups 3 (moderate dysbiosis) and 4 (severe dysbiosis). Furthermore, there was an increased trend for cell death from group 1–4, reflecting cell damage also described in the 3D model of human cervix exposed to *A. vaginae* (42, 43). Another study evaluated lactic acid treatment of cervicovaginal epithelial cell lines, with physiologically concentrations of short chain fatty acid (SCFA) and described the induction of an anti-inflammatory environment (increase of IL-1RA). Moreover, lactic acid and SCFA mixture significantly decreased the expression of IL-6, IL-8, CXCL10 and CCL20 upon stimulation with TLR1/2 and TLR3 agonists. On the contrary, higher concentration of SCFA, compared to lactic acid, induced an increased expression of TNFα and a decreased expression of CCL5 and CXCL10 upon TLR stimulation, suggesting that elevated SCFA might be responsible for the increased inflammation observed in the vagina of BV+ women (44).

### 2.2. Cell recruitment and phenotype

The vaginal microbiota has also an impact on immune cell recruitment and phenotype (Figure 1). *L. acidophilus* administration to BV+ mouse model colonized by *G. vaginalis* efficiently inhibits NF-κB pathways as well as IL-1β, IL-17, TNFα and increases IL-10 and Foxp3 expression. Moreover, the *Lactobacillus* species inhibited, *in vitro*, the differentiation of mice splenocytes into Th17 cells but increased their differentiation into Tregs (45). Similarly, *in vitro* experiment described that *G. vaginalis* was able to increase the production of IL-17, whereas *Lactobacillus* spp. induced a higher level of IFNγ. The authors suggested that BV associated bacteria might induce a Th17 response that may impair antiviral response and facilitate disease progression (46). Women with a diverse vaginal microbiota with low *Lactobacillus* spp. abundance (CST-IV) had a 17-fold increase in HIV-1 target cells in the FRT with elevated CCL3 and CCL5, which attracts CCR5+ cells. CVL of women with CST-IV had higher levels of IL-1α, TNFα, IL-8, IL-12p70, IFNγ, IL10, IL-17 and IL-17 inducing cytokines IL-23 and IL-1β. Moreover, vaginal inoculation of *P. bivia* in germ free mice promoted the recruitment in the FRT of CCR5^+^ CD4^+^ T cells compared to mice inoculated with *L. crispatus* (47). A. Semaganda et al. suggested that Treg might be important in the regulation of inflammation induced by BV associated bacteria but do not affect directly the presence of BV associated bacteria (48). Cervical cytobrushes of HIV exposed seronegative women (HESN) exhibited an increased frequency of NK cells, CXCR5^+^ CD8^+^ T cells, follicular T cells compared to HIV unexposed healthy women. In terms of vaginal microbiota, the HESN cohort exhibits a higher bacterial diversity but with an increased abundance of *L. crispatus*, *L. gasseri* and *L. iners* compared to the healthy cohort. These parameters may explain the protection of HESN from HIV-1 infection (49). In contrast, Lennard et al. did not find any differences in the frequency of CD4^+^ CCR5^+^ cells in cervical cytobrushes of BV+ women, suggesting the implication of other factors in the increased susceptibility of BV+ women to HIV-1 (37). No differences were detected in CD11^+^ DC or CD14^+^ monocytes and macrophages frequency in cervical cytobrushes when comparing BV+ and BV− women. However, in women with highly diverse microbiota, there were gene upregulations for NF-κB, TLR, NOD like receptor and TNFα signaling pathways in cervical APCs, most likely with LPS acting as the upstream regulator. CD4^+^ T cells frequency, obtained from cervical cytobrushes for those women, were higher and cells were more activated (HLA-DR^+^, CD38^+^, CCR5^+^) (17). RNA sequencing of isolated cell populations from cervical cytobrushes highlight that antigen presenting cells (APC) is the main cell population involved in FRT inflammation. The authors detected that gene upregulation in APC isolated from dysbiotic patients are involved in cell growth suppression, apoptosis, inflammatory pathways, neoplastic suppression and pro-proliferative pathways (50). Ectocervical tissues of BV− women harbored higher number of CD45^+^ cells, including CD3^+^, CD8^+^ and HLA-DR^+^ cells. Nevertheless, BV+ women had higher number of CD4^+^ and CCR5^+^ cells (51).

Overall, polymicrobial vaginal microbiota composition has been associated with an increased inflammation (higher pro-inflammatory cytokines production, upregulation of signaling pathways involved in inflammation, cell recruitment), epithelial barrier disruption, mucus impairment, cytoskeleton alteration and increased cell deaths (Figure 1; Table 1).

**Table 1:** Effects of bacterial vaginosis on the immune responses.

Factors influenced by the vaginal microbiota	Effects of BV	References
Soluble factors	(+) IL-1α, IL-1β, TNFα, IFNγ, IL-8, IL-12-p70, IL-4, IL-10, FLT-3L, G-CSF, GM-CSF, PDGF, CCL4, IL-36 (+) Antimicrobial peptides (psoriasin, calprotectin)	(17, 38, 40–42)
Cell recruitment	(+) Th17, CCR5+ CD4+, HLA-DR+ CD38+ CCR5+ (−) Treg	(17, 45, 47)
Other effects (metabolisms, mucus…)	(+) Inflammation pathways (NF-κB, TLR, NOD, TNFα…) (+) Epithelial barrier disruption (+) Mucus impairment (MUC5B, MUC5AC) (+) Cell deaths	(17, 42, 50)

(+) Increase; (−) decrease.

### 2.3. Neutrophils and microbiota

#### 2.3.1. Neutrophils in the FRT

Neutrophils are key mediators of inflammation and are present in all the compartments of the FRT (Figure 2). The highest presence of neutrophils is in fallopian tubes and this number decreases from the upper to the lower tract (52). In humans, neutrophils in fallopian tubes have been described to be phenotypically and functionally different from the neutrophils in the blood. Indeed, they expressed lower level of CD66b, CD62L, IL-8 receptor (CXCR1, CXCR2) and higher level of bacterial product receptor, CD64, HLA-DR, IFNγ, TNFα, IL-12 and VEGF compared to blood neutrophils (53). In the uterus, a strong increase of IL-8 before the menstruation leads to a significant recruitment of neutrophils, mainly through CXCR2 signaling pathway (54, 55). Their role during menstruation is to allow the disruption of endometrial tissue through the release of elastase, which activates extracellular matrix-metalloproteinases and increases immune defense (4). In a mouse model, neutrophil depletion induces a blockade of the estrous cycle due to a dysregulation of serum steroid hormone levels, suggesting an important role of neutrophils in regulating hormone levels (56). Estradiol treatment of mice infected with *Candida albicans* blocked neutrophil migration to the vagina and accumulated them in the ectocervix and fornix. This phenomenon is mediated by an altered expression of CD44 and CD47 expression of FRT epithelial cells enabling neutrophils to migrate to the vagina. On the contrary, progesterone treatment facilitates neutrophil migration and killing. These data suggested that neutrophil migration is dependent on sex hormones. Indeed, during the ovulation, neutrophils are not present in the vaginal lumen to facilitate the passage of sperm cells, although it may lead to vaginal infections (57).

Recently, we have described the phenotype of neutrophils in cervicovaginal cytobrushes obtained from female macaques and shown that they are different from neutrophils in the blood. We used several surface markers to characterize the priming (CD11b, CD62L), activation (CD11b, CD32a) or maturation (CD10, CD101) status of the neutrophils. We observed three main cervicovaginal neutrophil populations: CD11b^high^ CD101^+^ CD10^+^ CD32a^+^ (mature/activated), CD11b^high^ CD101^+^ CD10^−^ CD32a^+^ (immature/activated) and CD11b^low^ CD101^low^ CD10^−^ CD32a^−^ (pre-neutrophil like). The proportion of those populations varied during the menstrual cycle. During menstruation, there was an increase of CD11b^high^ CD101^+^ CD10^+^ CD32a^+^ subset of neutrophils, which expressed higher levels of CD62L than other populations of cervicovaginal neutrophils (58).

Very few is known about the relationship between the vaginal microbiota and local neutrophils in the FRT in contrast to what is described in the gut (59). We will now discuss how the relationships between the microbiota and neutrophils are important to modulate the inflammation at the level of the FRT.

#### 2.3.2. Regulation of the microbiota by neutrophils

##### 2.3.2.1. Neutrophil recruitment and activation

Neutrophils are among the most abundant immune cells in the vaginal lumen (58, 60). They are rapidly recruited to the mucosa during the inflammation process and thus are crucial for maintaining the balance between commensals and pathogens at the level of the vaginal microbiota. In BV+ women, elevated vaginal level of IL-8 and IL-1b has been correlated to the presence of pathogenic bacteria, leading to a high neutrophil count (61). On the contrary, many probiotic strains of *Lactobacillus* have been shown to induce lower TLR response compared to pathogenic strains *in vitro* (62, 63). This may lead to an activation of neutrophils that depends on the vaginal microbiota composition as demonstrated in the gut (64, 65).

##### 2.3.2.2. Regulation of neutrophil function

Neutrophils can elicit a vast antimicrobial response and impact the microbiota composition. For example, upon pathogen recognition, neutrophils can produce various AMPs and proteins, through granule secretion, and those AMPs are important for the containment of the microbiota (66, 67).

Reactive oxygen species (ROS) are produced by local neutrophils in the FRT (68), and contribute in the one hand to enhance the oxidative stress induced by *Lactobacillus* H_2_O_2_ production, leading to killing or restriction of pathogen growth (69), and on the second hand, they mediate neutrophil extracellular trap (NETs) production. NETs are networks composed of DNA and neutrophil proteins that can bind pathogens and facilitate their killing by neutrophils (70). In the FRT, exaggerated NET formation might lead to infertility since NETs can also damage endogenous cells. The vaginal microbiota could also have an indirect effect on NET release by neutrophils. Indeed, cervical mucins have been described to have anti-inflammatory properties, leading to the inhibition of NET release in the FRT (71). The vaginal microbiota composition is able to regulate the cervicovaginal mucus (CVM). Indeed, anaerobic bacteria can degrade the CVM (72), and thus could enhance indirectly NET production through the lifting of NET release inhibition induced by mucins.

In conclusion, neutrophils are important to control the vaginal microbiota and respond to pathogens, but there is also a need to maintain a balance between recruitment/activation of the neutrophils and tolerance of the microbiota to prevent various reproductive health issues including infertility. The microbiota also produces numerous components and metabolites that can have an impact on neutrophil production, maturation and functions.

#### 2.3.3. Regulation of neutrophils by the microbiota

##### 2.3.3.1. Regulation of neutrophil production and maturation

Several studies have shown that the microbiota is essential in the production of neutrophils in the gut but nothing has been reported yet in the FRT (73, 74).

Metronidazole treatment of BV+ women lead to an increased production of GM-CSF which appeared to be associated with the abundance of *L. crispatus* (75). Although the authors did not monitor hematopoietic cell proliferation and differentiation, this result could suggest that the vaginal microbiota could be involved in neutrophil production.

##### 2.3.3.2. Regulation of neutrophil circulation

The circadian circulation of neutrophils is also influenced by the microbiota. In germ free mice, *Klebsiella pneumoniae* lung infection leads to a limited neutrophil mobilization favoring the pathogen growth (76). Microbiota depletion in mice induces a reduced number of aged neutrophils (CD62L^−^, CXCR4^+^) in the blood, and exhibit significant reductions in neutrophil adhesion/Mac-1 activation compared to untreated mice, leading to a retention of neutrophils in the circulation (77). Myd88, TLR2 and TLR4 knockout mice also exhibit less aged neutrophils, suggesting a role of the microbiota in priming neutrophils (77). Another paper has shown that administration of *Lactobacillus acidophilus* induced a decrease in pro-inflammatory cytokines (IL-8, TNFα, IL-6), immunoregulative cytokines (IL-4, IL-10), neutrophil and macrophage infiltrates as well as a reduced injury severity in rats suffering from colitis (78). The microbiota could have a local effect on epithelial cells leading to the recruitment of neutrophils, and a distal effect through secretion of metabolites that diffuse into the blood. Indeed, it has been shown that low concentrations of TLR ligands such as LPS can be detected in the bone marrow and induced neutrophil trafficking into the blood (79). It has also been shown that bacteria from the gut microbiota can produce short-chain fatty acids (SCFA), and could have an impact on neutrophil recruitment at the site of inflammation (80).

##### 2.3.3.3. Regulation of neutrophil functions

The microbiota can have a direct or indirect effect (for example, by modulating the inflammation) on neutrophil functions. Neutrophils isolated from germ-free or antibiotics treated mice exhibit decreased antimicrobial functions, such as a decreased in myeloperoxidase activity (81), in phagocytic capacity (82) or a reduction in NET formation (77).

Metabolites such as short chain fatty acid, secondary bile acid lithocholic acid or histamine produced by the microbiota, modify ROS production or phagocytosis activity in neutrophils (59, 83). Butyrate and propionate inhibit pro-inflammatory cytokines (TNFα and cytokine induced neutrophil chemoattractant-2) and nitric oxide production by LPS stimulated neutrophils (rat model). Moreover, those SCFA are also able to inhibit HDAC and NF-κB activation, which might explained the decrease in pro-inflammatory cytokines (84). In a mouse model for endometritis, a dysbiosis was induced in the gut microbiota, and uterine colonization of *S. aureus* led to endometrial inflammation, associated with increased pathogen load in the uteri of the mice with gut microbiota-dysbiosis associated with a low phagocytic capacity and responsiveness of neutrophils. A fecal microbiota transplantation was then performed, and those effects were reversed. The authors demonstrated that protective effect was due to SCFAs present in the feces of mice and that diffuse into the blood but also in uterine tissues.

Most of the mechanisms for neutrophil regulation were observed in the gut, but might also be important in the vagina in terms of neutrophil priming and function for instance. *Lactobacillus* impact on neutrophils might also occurred in the vagina. Interestingly, R. Cheu et al. have observed an activation of cervicovaginal neutrophils, as well as a delay in neutrophils apoptosis leading to neutrophil accumulation within the vagina of women suffering from bacterial vaginosis (Keystone 2018).

## 3. Vaginal microbiota and STI

### 3.1. Interaction between STI and the vaginal microbiota

Numerous studies evaluating bacterial vaginosis either by Nugent score, culture or Amsel criteria described association between STI acquisition and BV (85–87).

#### 3.1.1. Bacterial STI including *Chlamydia trachomatis* and *Neisseria gonorrhoeae*

The vaginal microbiota of adolescent girls or women dominated by diverse anaerobic bacteria (such as *G. vaginalis*, *Porphyromonas somerae*, *Corynebacterium urealyticum*, *Dialister* spp., *Megasphaera*, *A. vaginae*, *Prevotella disiens*) or *L. iners* are more likely to be infected by bacterial STI agents such as *Chlamydia trachomatis* (CT), compared to women with a vaginal microbiota dominated by *L. crispatus* (88). Similar bacteria are increased in women suffering from chlamydiosis or candidiasis (*Gardnerella*, *Prevotella*, *Faecalibacterium*, *Megasphaera*, *Roseburia* and *Atopobium*) in addition to a *Lactobacillus* spp. depletion (89). BV+ women were at increased risk (3.4 times) to acquire *N. gonorrhoea* and CT, compared to women colonized with hydrogen peroxide producing *Lactobacillus* (29). Interestingly, *N. gonorrhoeae* asymptomatic patients had more frequently a *Lactobacillus* dominant microbiota and a lower bacterial diversity compared to symptomatic patients, suggesting a link between symptoms and the vaginal microbiota (90). Furthermore, women with tubal infertility and CT infection had a vaginal microbiota dominated by *L. iners* and a decrease of other *Lactobacillus* spp., *Bifidobacterium*, *Enterobacter*, *Atopobium* and *Streptococcus* (91). In opposition to CT infected women, *L. iners* was observed to be decreased in *Trichomonas vaginalis* infected women whereas *Streptococcus agalactiae*, *Prevotella bivia*, *Sneathia sanguinegens* and *Gemella asaccharolytica* were increased (92). Presence of *Prevotella amnii* and *S. sanguinegens* have been associated with a 2 fold increased risk for *T. vaginalis* infection (93). Also, the co-occurrence of *Gardnerella*, *Peptoniphilus*, *Dialister*, *Atopobium*, *Parvimonas* and *Metamycoplasma hominis* in CT positive women may lead to biofilm formation (94). *Mycoplasma hominis* and *Ureaplasma urealyticum* are often detected in the vagina/cervix of sexually active women with BV (95–97).

#### 3.1.2. Human papilloma virus (HPV)

Microbial variation is detected in women with high risk HPV infection, characterized by a decrease of *Lactobacillus* spp., *Veillonella*, *Sneathia*, *Sporolactobacillus* and an increase of *Gardnerella*, *Prevotella*, *Dialister*, *Slackia*, *Actinomyces*, *Porphyromonas*, *Peptoniphilus*, *Anaerococcus*, *Peptostreptococcus*, *Streptococcus*, *Ureaplasma*, *Megasphaera* and *Mycoplasma* (98). In addition, cervicovaginal swabs of HPV^+^ women exhibit higher levels of biogenic amine and phospholipid compared to HPV- women (99). In a meta-analysis performed by Tamarelle et al., a vaginal microbiota dominated by *Lactobacillus* was described to have a protective role against HPV and CT (100). In addition, a higher susceptibility to HPV was determined in BV+ women (101). HPV infection was shown to reduce innate peptide expression including defense peptides expressed by the vaginal or uterine epithelium. Moreover, *Lactobacillus* was shown to be able to degrade and use defense peptide as an amino acid source to survive. Therefore, the authors hypothesized that HPV infection, by reducing defense peptide availability, lead to a reduction of *Lactobacillus* and an increase of BV associated bacteria that will promote HPV disease progression (101). In HPV^+^ women that cleared the infection and had a cervical intraepithelial neoplasia removal, an increased abundance of *L. crispatus* was observed compared to women with a persistent infection. Interestingly, *G. vaginalis* was present in all women suffering from persistent infection following surgical treatment (102). Furthermore, during HPV persistence and post-clearance, *G. vaginalis* abundance was increased and associated with a higher cytokine concentration (103).

#### 3.1.3. HIV-1

A higher vaginal bacterial diversity was observed in HIV-1+ women and *Dialister*, *Gemella asaccharolytica*, *Eggerthella*, *Parvimonas micra*, *M. hominis*, *Sneathia*, *Megasphaera* and *Leptotrichia amnionii* were associated with higher risk of HIV-1 acquisition (104). In the FRESH cohort, women with high vaginal microbiota diversity and a low abundance of *Lactobacillus* (CST-IV) had a 4-fold increased risk for HIV-1 acquisition compared to women with a microbiota dominated by *L. crispatus* (CST-I). Moreover, the authors observed that none of the women with *L. crispatus* dominated microbiota acquired HIV-1 (47). In a vaginal mucosal culture model, the cervicovaginal fluids from women with CST-I inhibited HIV-1 infection, compared to women with CST-IV. HIV-1 infection was even enhanced when the *in vitro* model was cultured with vaginal fluids of women with CST-IV containing high abundance of *Ruminococcaceae* spp., *S. sanguinegens*, *A. vaginae* and *Aerococcus* spp. (105).

Overall, a higher bacterial diversity of the vaginal microbiota, associated with an increase of BV associated bacteria and a decrease of *Lactobacillus* spp. (except *L. iners*) abundance induce a higher susceptibility to various viral, bacterial and fungal STI (Figure 2; Table 2).

**Table 2:** Vaginal microbiota composition observed in STI patients or bacteria that are able to enhance pathogen acquisition.

Pathogens	Vaginal microbiota composition in infected individuals	Bacteria that enhance pathogen acquisition	References
*Chlamydia trachomatis*	(+) *Gardnerella*, *Prevotella*, *Faecalibacterium*, *Megasphaera*, *Roseburia* and *Atopobium*, *L. iners* (−) *Lactobacillus* spp., *Bifodobacterium*, *Atopobium*, *Streptococcus*	*G. vaginalis*, *Porphyromonas somerae*, *Corynebacterium urealyticum*, *Dialister* spp., *Megasphaera*, *A. vaginae*, *Prevotella disiens* and *L. iners*	(88, 89, 91)
*Trichomonas vaginalis*	(+) *Streptococcus agalactiae*, *Prevotella bivia*, *Sneathia sanguinegens*, *Gemella asacharolytica* (−) *Lactobacillus* spp.	*Prevotella amnii*, *Sneathia sanguinegens*	(92, 93)
HPV	(+) *G. vaginalis*, *Eggerthella*, *Atopobium* spp. (other than *A. vaginae*), *Dialister* spp., *Veillonellacea*, *Aerococcus christensenii*, *Peptoniphilus asaccharolyticus*	*G. vaginalis*, *Prevotella*, *Dialister*, *Slackia*, *Actinomyces*, *Porphyromonas*, *Peptoniphilus*, *Anaerococcus*, *Peptostreptococcus*	(98, 99, 103)
HIV-1	(+) *Dialister*, *Gemella*, *asacharolytica*, *eggerthella*, *Parvimonas micra*, *Mycoplasma hominis*, *Sneathia*, *Megasphaera*, *Leptotrichia amnionii*, (−) *Lactobacillus crispatus*	*Ruminococcaceae* spp., *Sneathia sanguinegens*, *Atopobium vaginae*, *Aerococcus* spp.	(47, 104, 105)

(+) Increase; (−) decrease.

#### 3.1.4. Co-infections

Co-infections within the FRT are frequent; indeed, a pre-existing infection can facilitate the acquisition of a second STI by modifying the local environment. Numerous epidemiology studies described that women suffering from an STI such as CT, Human Simplex Virus type 2 (HSV-2), *Neisseria gonorrhoeae*, *Treponema pallidum*, or *Trichomonas vaginalis* have an increased risk of HIV-1 acquisition (106–108). Several mechanisms might be involved within the FRT: for instance, CT infection may facilitate HIV-1 acquisition via cytokine production, leading to the recruitment of HIV-1 target cells, higher expression of HIV-1 coreceptors CXCR4 or CCR5, or epithelial cell disruption allowing HIV-1 cell free virus to cross the epithelial barrier… (109–111). Interestingly, vaginal microbiota of CT/HPV or HSV-2/HIV-1 co-infected women have an increased bacterial diversity (112, 113). Furthermore, Borgogna et al. described a distinct metabolic profile between uninfected and CT infected or CT/*Mycoplasma genitalium* coinfected women (30). The interplay between vaginal microbiota, metabolic signatures and FRT inflammation during an STI needs to be better addressed in order to understand how those modifications may facilitate the acquisition of a second STI.

### 3.2. Crosstalk between neutrophils and the microbiota during STI exposition

During STI exposition, crosstalk between neutrophils and the microbiota can lead to various outcome in terms of STI acquisition and disease progression (Figure 2). Hensley-McBain et al. have shown that neutrophil accumulation in colorectal biopsies of HIV-1 infected ART treated patients is due to an increase of functional neutrophil survival. The authors observed an altered ratio of *Lactobacillus*/*Prevotella* in HIV-1 positive individuals, which correlated with neutrophil survival in colorectal tissue from the same patient. *In vitro*, *Prevotella*, *Bacteroides fragilis* and *Ruminococcus bromii* increased neutrophil survival compared to *Lactobacillus plantarum* and *Lactobacillus rhamnosus* (114). Neutrophils are known for being involved in protection against various STI. However, in neutrophils isolated from HIV-1 positive individuals, PRR expression is reduced, and upon TLR stimulation, neutrophils exhibit a reduced cytokine expression (115). During the chronic phase, a dysbiosis occurs (characterized by a *Gammaproteobacteria* enrichment and a *Lachnospiraceae*/*Ruminococcaceae* depletion) (116). The hyporesponsiveness of neutrophils, combined with this dysbiosis could be involved in disease progression, but usually tissue neutrophils are often poorly analyzed, and more studies are necessary to fully understand the role of neutrophils at mucosal surfaces and their association with STI risk disease progression (117).

In the FRT, various STI agents induce neutrophil activation and antimicrobial function, including NET production, which prevents several STI acquisition notably during the early phase of infection (68, 118). However, bacterial STI have also developed mechanisms to counteract neutrophil-mediated immunity. For example, *N. gonorrhea* and *C. trachomatis* resists neutrophil cytotoxic activity by several mechanisms, including inhibition of NET formation (119–121). However, up to date, there is no study on the impact of the vaginal microbiota composition on neutrophil-mediated immunity during bacterial STI. Probiotics could help maintaining an environment suitable for a balanced neutrophil recruitment and antimicrobial functions. For example, *Lactobacillus* have been used in the gut to reduce infection severity linked to inflammation. Neutrophil accumulation is reduced upon *Lactobacillus* treatment. Oral administration of *L. plantarum* CIRM653 before *K. pneumoniae* infection in mice, induced an immunosuppressive Treg response and decreased the number of immune cells (macrophages and neutrophils) and pro-inflammatory cytokines (IL-6 and TNFα) compared to naïve mice (122).

In conclusion, neutrophils are present at steady state in the FRT, but can also be rapidly recruited upon infection. Thus, they might have various roles in STI acquisition, notably depending on the vaginal microbiota composition, which is an important modulator of the local inflammation.

### 3.3. Mechanisms of action of the vaginal microbiota

**Figure 3:**
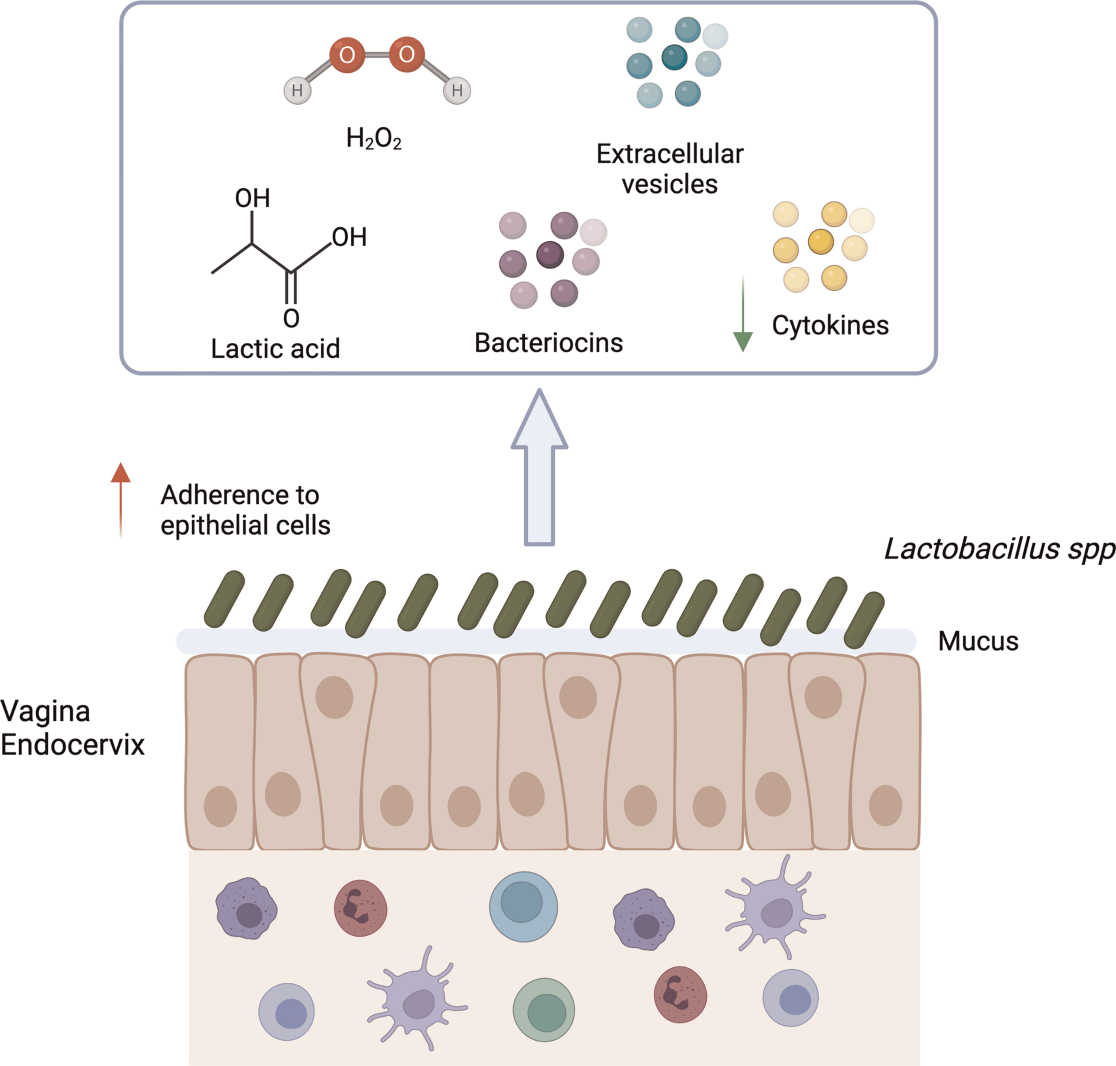
Mechanisms of action associated with *Lactobacillus* spp. presence. *Lactobacillus* spp. decrease the risk of sexually transmitted infection acquisition by several mechanisms: (1) Aggregation to epithelial cells protecting the cells from pathogen; (2) Maintenance of mucus integrity; (3) Low inflammation (low production of cytokine and immune cells recruitment); (4) Production of factors (lactic acid, bacteriocins, H_2_O_2_, extracellular vesicles) involved in pathogen inhibition. Created with BioRender.com.

*Lactobacillus* have been proposed as probiotic agents to be used against pathogenic microorganisms in the vagina. They can enter in competition with pathogens for attachment sites on epithelial cells, co-aggregate with pathogens and produce antimicrobial compounds. Indeed, *Lactobacillus* can produce: (1) biofilm allowing to mask epithelial cell receptors, adhesion to mucins and epithelial cells (123, 124) (2) lactic acid that inhibits pathogen growth (3) bacteriocins and bacteriostatic agents (Figure 3).

*Lactobacillus* are able to decrease bacterial infection such as CT either by aggregating to extracellular elementary body (EB), by competition for epithelial cells attachment or through lactic acid production that inactivate CT (125, 126). *L. crispatus* strains seem to have the best anti-CT effect (127). *In vitro*, *L. crispatus* treatment of a CT infected epithelial cell line (J774) decreased the production of IL-6, IL-8 and TNFα and induced the production of IL-10 (128). In addition, IL-1α, IL-1β and TNFα were strongly associated with CT infection in women that had a change in microbial communities (17). Pre-colonisation of epithelial cells with *L. crispatus* or *L. crispatus* enolase/glutamine synthetase decreased the adhesion and invasion of *N. gonorrhoea* probably by entering in competition for epithelial cell attachment. In addition, the expression of TNFα and CCL20 was also decreased in the pre-colonisation model (129). *Lactobacillus* spp. was observed to affect MyD88 and NF-κB signalling pathways. *L. johnsonii* was able to activate the TLR2/TLR4 mediated NF-κB pathway (130). In addition, pretreatment of cells with *L. rhamnosus* before *E. coli* infection suppressed Myd88, NF-κB, TICAM2 expression associated with a decrease of IL-1β, IL-18, IL-6, IL-8, IL-10, IFNβ and TNFα (131). Many *Lactobacillus* spp. were described to be able to inhibit *T. vaginalis* adhesion on epithelial cells (132). Another anti-CT mechanism could result from a decreased availability of nutrients (such as glucose) in the environment, thanks to its consumption by *Lactobacillus* spp. (127). Annelot C. Breedvelt et al. demonstrated that women with a *L. crispatus* dominated microbiota had a higher level of bacterial IgA coating compared to non-*L. crispatus* dominated microbiota. The authors suggested that a higher level of unbound bacteria with IgA in non-*L. crispatus* dominated microbiota might be associated with inflammation, and therefore a higher susceptibility to infection (133).

#### 3.3.1. Effect of H_2_O_2_ on pathogens

H_2_O_2_ was described as a potent antimicrobial factor produced by *Lactobacillus*, however, under the hypoxic conditions found in the vagina, *Lactobacillus* production of H_2_O_2_ is undetectable (134, 135). Moreover, physiological concentrations of H_2_O_2_ are not sufficient to have a bactericidal effect on pathogens, suggesting that the main antimicrobial agent produced by *Lactobacillus* is lactic acid (135).

#### 3.3.2. Effect of lactic acid on pathogens

Vaginal lactic acid exists as D and L-isomers. Epithelial cells can only produce L-lactic acid, *L. crispatus* and *L. gasseri* are able to produce both isomers, *L. jensenii* only produces D-lactic acid and *L. iners* only produces L-lactic acid (136). Lactic acid production acidifies the vagina, but *in vitro*, when the pH reaches 3.2–4.8, *Lactobacillus* stop their growth and acid lactic production (137). *L. iners* colonization has been linked to a higher vaginal pH compared to other species of *Lactobacillus* (138). Matrix metalloproteinase-8, which is able to degrade the cervical plug, is down regulated by D-lactic acid trough decrease of extracellular metalloproteinase inducer (EMMPRIN), thus inhibiting pathogen migration to the uterus (136).

Lactic acid has an important antimicrobial effect in the vagina. *L. crispatus* pre-colonisation of porcine vaginal mucosa model inhibits *N. gonorrhoeae* growth mainly through lactic acid production (139). A significant association between a low HIV-1 infectivity and a high percentage of D- and L-lactic acid, which also correlated with a high relative abundance of *Lactobacillus* spp. (particularly *L. crispatus*) has been reported (140). Moreover, cervicovaginal mucus with a high concentration in D-lactic acid collected from women demonstrated a better HIV-1 trapping. These mechanisms seem to be mediated through hydrogen bonds between surface carboxyl groups of the virus and host-derived envelope glycolipids or glycoproteins. Furthermore, CVM that trapped HIV-1 were generally associated with a *L. crispatus* dominant microbiota whereas those that failed to trap HIV-1 had either a *L. iners* dominant microbiota or a significant abundance of *G. vaginalis* (141, 142). On the contrary, Nahui Palomino et al. have shown that L-lactic acid but not D-lactic acid were the main contributors for HIV-1 inhibition on cervicovaginal explants. The authors also demonstrated that *Lactobacillus* were able to bind to HIV-1 virions, thus decreasing HIV-1 infection (143).

#### 3.3.3. Effect of bacteriocins on pathogens

Bacteriocins are antimicrobial peptides or proteins secreted by some but not all vaginal *Lactobacillus* strains (144, 145). Bacteriocins are divided into two classes: class I bacteriocins (lantibiotics) are lanthionine-containing peptides, whereas class II bacteriocins do not contain lanthionine (146). Class I bacteriocins are active through the formation of pores and efflux of small metabolites from sensitive cells or through enzyme inhibition. The majority of class II bacteriocins are active by inducing membrane permeabilization and the subsequent leakage of molecules from target bacteria. Bacteriocins isolated from vaginal *Lactobacilli* were shown to inhibit the growth of various microorganisms including *Klebsiella*, *Staphylococcus aureus*, *Escherichia coli*, *Enterococcus faecalis*, *Candida parapsilosis* or *G. vaginalis* (144, 145). Bacteriocins are also active against STI agents such as *N. gonorrhoeae* (147). In a recent study, a *Lactobacillus* strain was even shown to selectively inhibit 100% of *L. iners* strains tested through two different class IIb bacteriocins: gassericin K7A and gassericin K7B (148). Among other strains of the vaginal microbiota including *L. crispatus*, *L. jensenii*, and *L. gasseri*, only few of them (<20%) were also inhibited by these bacteriocins. *In vitro* studies showed that bacteriocin production was not constitutive but only occurred in specific conditions: for example through co-cultivation with several other Gram-positive strains (149) or during the exponential growth phase of *Lactobacilli* (150), when bacteriocin production was probably being enhanced by autoinduction (149). The ability of various *Lactobacillus* strains to produce bacteriocins that efficiently inhibit vaginal pathogens may allow their use as therapeutic agents for the treatment and prevention of urogenital disorders and could be an alternative to conventional antibiotic therapy (150).

#### 3.3.4. Effect of extracellular vesicles on pathogens

A recent study has linked extracellular vesicles (EV) produced by *Lactobacillus* and protection against HIV-1. EV are produced by Gram (+) and Gram (−) bacteria. In Gram (−), they have been described to be produced by the pinching off of the outer membrane of the bacteria. However, the mechanism implied in EV production in Gram (+) bacteria is not yet determined (151). EV produced by *Lactobacillus* spp. isolated from women inhibit HIV-1 replication in CD4^+^ T cells, human tonsillar and cervicovaginal tissues in a dose dependent manner. HIV-1 inhibiting EV were associated with more amino acids, and the expression of several proteins such as enolase and elongation factor Tu. Those proteins were reported to play a role in adhesion to epithelial cells (152). Interestingly, Paolo E. Costantini et al. described that other Gram (+) bacteria, such as *S. aureus*, *G. vaginalis*, *Enterococcus faecium* and *E. faecalis* were able to produce EV that protect against HIV-1 infection. The mechanism associated with this protection is through a steric hindrance of gp120. Moreover, several proteins present on EV surface are involved in the anti-viral effect (153). This mechanism seems to be not exclusive of *Lactobacillus* spp., therefore evaluation and comparison between species/strains need to be addressed to understand if EV produced by *Lactobacillus* spp. have a better HIV-1 inhibitory effect compared to EV produced by anaerobic bacteria.

#### 3.3.5. Impact of indole production by the vaginal microbiota during the CT infection

The vaginal microbiota can also impact the pathogenesis of STI infection. For example, upon CT infection, IFNγ production induces the activation of the enzyme IDO1 which depletes the tryptophan pool within the epithelial cells into kynurenine. *In vitro*, pretreatment with IFNγ of CT (svD) infected epithelial cell induces the depletion of the tryptophan pool, leading to no infection of the cells. Supernatant of indole producing bacteria such as *Prevotella intermedia* and *Prevotella nigrescens* promotes CT infection in presence of IFNγ (154). Interestingly, *in vitro*, IFNγ induced a persistent CT phenotype which was described to have a distinct proteome profile compared to EB and reticulate body (RB) with a high expression of the tryptophan operon TrpA and TrpB (155). These proteins are essential for tryptophan synthesis using indole produced by anaerobic bacteria present in the FRT (156). Ratios of kynurenine/tryptophan are higher in vaginal fluids of CT infected women, and correlated with high abundance of *Streptococcus* spp. and *Peptoniphilus* spp. Moreover, 94% of the samples of CT infected women exhibited a high abundance of indole producing bacteria such as *Porphyromonas asacharolytica*, *Propionibacterium acnes*, *Fusobacterium nucleatum*, *E. faecalis*, *Peptoniphilus harei* and *E. coli* (157). CT can retain a subset of genes involved in tryptophan synthesis (*trpA*, *trpB* and *trpR* genes) and use them to synthesize tryptophan from indole, leading to CT growth and infection (158).

#### 3.3.6. Impact of the vaginal microbiota on antiviral drugs

Vaginal microbiota composition has been linked to antiretroviral microbicide gel protection against HIV-1. The efficacy of tenofovir (TFV) to decrease HIV-1 infection was reduced in culture colonized by bacteria from vaginal fluids of women with a diverse microbiota (105). In the CAPRISA study, TFV gel reduced HIV-1 incidence by 61% in women with a vaginal microbiota dominated by *Lactobacillus*, but only by 18% in women with a non-*Lactobacillus* dominant microbiota. Furthermore, TFV concentration in CVL negatively correlated with BV associated bacteria such as *G. vaginalis* and *Prevotella*. *G. vaginalis* and to a lesser extend *Prevotella* species, are able to rapidly metabolized TFV before drug uptake by target cells. The mechanism involves the production of adenine by cleavage of the side chain component of TFV (oxy-methylphosphonic acid) (159). Moreover, CVL, collected from women suffering from BV, were able to degrade TFV and dapirivine (DPV) but not tenofovir alafenamide (TAF). TAF does not seems to be impacted thanks to a faster internalization in target cells (159, 160). These articles demonstrated the importance of *Lactobacillus* in preventing drug degradation by other species of bacteria, highlighting that microbial communities are critical for TFV and DPV efficacy (160). On the contrary, Taneva et al. suggest a more complex role of the microbiota in the modulation of topical antiretroviral efficacy. The authors observed adenine production in the culture medium, which is able to block endocytosis of TFV into human cells, leading to decrease protection against HIV-1. They also observed that TFV is transported and metabolized by *L. crispatus* but not *L. jensenii* and *L. iners*. The authors hypothesized that *L. crispatus* could be a reservoir that gradually release TFV into the vagina. Finally, they observed that the inhibitory effect of the vaginal microbiota was overcome at higher drug levels, suggesting that a sustained drug delivery could be sufficient to induce protection. However, a decrease in drug levels might lead to a decreased efficacy of antiretroviral due to adenine and high pH (161). Intravaginal rings could be used to induce a sustained delivery of drugs. DPV ring use in adolescents was not associated with cervicovaginal inflammation or microbiota changes, however, a significant increase in *L. crispatus* and a decrease of *L. iners* were observed when both arms were combined (placebo ring and DPV ring), suggesting a beneficial effect of ring use on the microbiota (162). TFV intravaginal ring in adults was also observed to have no impact on vaginal microbiota composition. Moreover, the authors described no effect of BV associated bacteria on TFV availability. The hypothesis was that sustained release of TFV by the ring is sufficient to abrogate the effect of BV associated bacteria (163). The efficacy of daily oral PrEP for HIV-1 prevention among women with highly diverse microbiota were not impacted, compared to women with a *Lactobacillus* dominant microbiota (164).

## 4. Non-human primate (NHP) models to study vaginal inflammation and microbiota in STI

**Figure 4:**
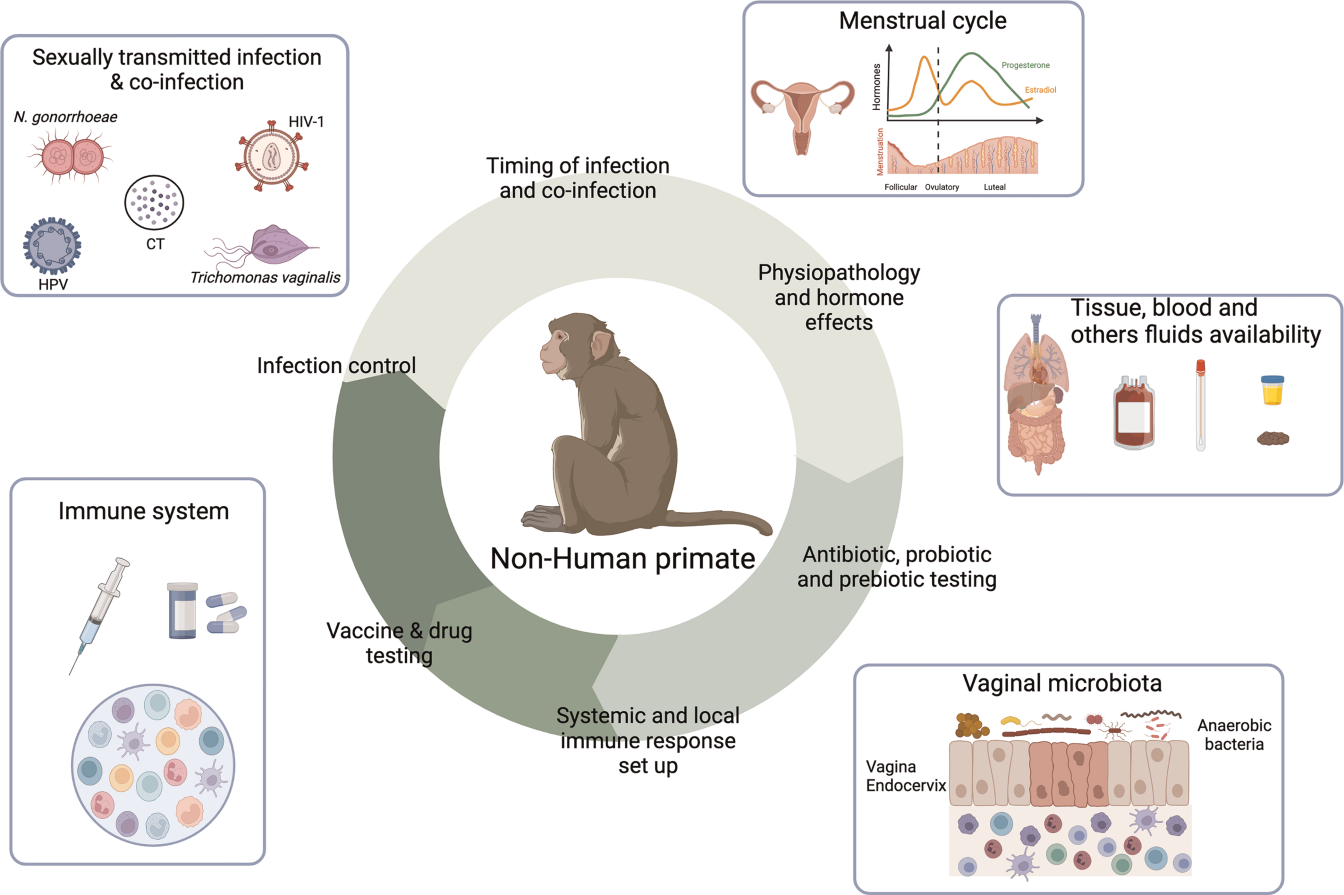
Contribution of the female NHP model in the understanding of the mechanisms involved in sexually transmitted infection acquisition and control. NHP and Human similarities include NHP susceptibility to several STI affecting Human, FRT anatomy, menstrual cycle, immune cells presence and vaginal microbiota composition. This model also allows the longitudinal collection of tissue samples, stool, blood or cervicovaginal fluids. Therefore, NHP can be used to study various parameters involved in STI acquisition and control including time and route of infection, susceptibility to infection/co-infection, physiopathology, hormone effects, in deep evaluation of the immune system, probiotic/prebiotic/vaccine/drug testing. Created with BioRender.com.

Animal models including mice, rat or non-human primates (NHP) have been widely used in biomedical research including for STI studies. Rodents are particularly used due to their numerous advantages including a low cost in terms of housing, easy breeding and numerous offsprings at each litter. Moreover, genetic engineering or microbiota modification can be easily performed in mice models for instance (165, 166). However, substantial differences can be highlighted between the mice models and humans. Divergence between humans and mice occurs between 65 and 75 million years ago (167, 168). Differences in terms of innate and adaptive immune responses can also be highlighted: the balance of leukocyte subsets (T and B cells), neutrophils and soluble factor production (cytokines and chemokines) are different for instance (168, 169). Moreover, FRT anatomy as well as hormonal secretion differ between humans and mice. For instance, the cervical epithelium is different between the two species (170). Furthermore, several infectious pathogens do not infect or recapitulate human pathology in mice (171). On the contrary, NHP have been described to be susceptible to wide range of human pathogens including viral (Simian Immunodeficiency virus, the simian analogue of HIV-1, ZIKA, Ebola, Chikungunya or Influenza) or bacterial infections (Periodontal disease, Tuberculosis, *Chlamydia trachomatis*) (171–174). They are essential to determine vaccine or drug safety/efficacy, immune responses against infectious pathogens that induce high mortality and tissue specific immune responses for instance. The NHP model can be used to study human STI by controlling various parameters such as the infectious dose, or the time of infection that are more difficult to assess in cohort studies (Figure 4). It is also possible to study the mechanisms involved during co-infections establishment, which is much more difficult in cohort studies.

### 4.1. Similarities between human and NHP

Humans and NHP are closely similar since their divergence occurs only between 6 and 7 million years ago (167). Therefore, it is not surprising to observe similitudes in terms of immune system, female reproductive tract and to a lower extend vaginal microbiota between the two species. Three species of macaques are commonly used: rhesus macaque (*Macaca mulatta*), cynomolgus macaque (*Macaca fascicularis*) and pigtail macaque (*Macaca nemestrina*) (175).

#### 4.1.1. Immune system

Immune cells including neutrophils, basophils, CD8^+^ and CD4^+^ T cells, B cells, NK cells, plasmacytoid DC, classical/intermediate or non-classical monocytes in the blood are observed in rhesus, cynomolgus macaques and humans and have similar surface markers (169, 176). For example, DC subsets represent less than 1% of cells in PBMC and express similar TLR in humans and rhesus macaques (RM). Both species have similar T cell subsets (naïve, central memory or effector memory) (177). However, some differences can be noticed between NHP and humans. Bjornson-Hooper et al., observed more CD4^+^ CD8^+^ double positive T cells, a lower ratio of classical/non classical DC, low expression of CD11c and CD16 on neutrophils and the CD8 expression on NK cells in the blood of cynomolgus or rhesus macaques compared to humans (169). The lower presence of B cells is also noticed in peripheral blood, mesenteric lymph node and spleen in cynomolgus macaques compared to humans (176).

#### 4.1.2. Female reproductive tract

The female reproductive tract of cynomolgus macaques is closely similar to the one of women in terms of morphology, endocrine system and menstrual cycle (178). The menstrual cycle in rhesus and cynomolgus macaques (28–32 days) is similar to the one observed in humans (28–30 days). The follicular phase lasts 12–14 days and the luteal phase around 14–16 days (179). Menstruation lasts between 1 and 8 days, but 85% of the females exhibit a menstruation cycle of only 3–5 days (180). RM exhibit seasonal variations in their sexual and gonadal activity (only active from October to March), contrary to marmoset and cynomolgus macaques (179).

Marlin et al. have characterized the immune cell in the vagina, cervix, uterus and fallopian tubes of cynomolgus macaques. The localization and phenotype of immune cells are similar in the human and cynomolgus macaque FRT (181).

These similarities between Humans and macaques allow a better characterization of the genital inflammation, the environmental factors impacting the inflammation and thus the development of strategies to modulate this inflammation. Indeed, in cynomolgus macaques, it is possible to test intervention strategies targeting immune cells or using microbicides to modulate the inflammation. Moreover, during those studies, it is possible to access various samples in longitudinal studies, including tissue sample for instance, that can be difficult to obtain in Humans (Figure 4).

#### 4.1.3. Vaginal microbiota

The vaginal microbiota of macaques exhibits low abundance of *Lactobacillus*, contrary to what is observed in eubiosis women. Many species of macaques harbor a diverse vaginal microbiota, composed of a wide range of bacteria including *Saccharofermentans*, *Campylobacter*, *Atopobacter*, *Thioreductor*, *Streptococcus* and interestingly, several BV associated bacteria such as *Gardnerella*, *Prevotella*, *Fusobacterium*, *Sneathia*, *Atopobium*, *Peptoniphilus*, *Dialister*, *Mobiluncus* and *Porphyromonas*. The vaginal pH is also higher than in humans, with a median value of 7 (182–186). *Lactobacillus* can be found in all species of macaques, but at a low abundance. In RM, most of the *Lactobacillus* detected are *L. johnsonii*, and few sequences correspond to *L. gasseri* and *L. salivarus*. A comparative study between humans and RM showed that oral, perianal and vaginal microbiota of RM were distinct from the human ones. Indeed, the vaginal microbiota was more diverse than the human vaginal microbiota. The vaginal microbiota of cynomolgus macaques is similar in terms of taxonomic composition and relative abundance to the one of women harboring a high vaginal microbiota diversity (CST-IV). It is also variable according to animals and correlates with hormonal variation during the menstrual cycle (186). The vaginal microbiota is more diverse during lactation and menstruation (187). More precisely, during the menstruation there is an increased concentration of pro-inflammatory cytokines, a higher abundance of mature/activated neutrophils that originate from the blood and vaginal microbiota fluctuation (58)

One hypothesis explaining the low abundance of *Lactobacillus* spp. in macaques concerns the levels of glycogen and lactic acid poorly present in the vaginal fluids of macaques compared to humans (188). Indeed, Miller et al. hypothesized that high level of starch in human diets have induced an increased amount of glycogen within the vagina, which in turn promotes the proliferation of *Lactobacillus* (189). Several research teams have developed macaque's model vaginally enriched with *Lactobacillus*, but were only partially successful (190, 191). The authors observed either a transient colonization with *L. crispatus* and/or no modification of cervicovaginal inflammation and pH. The diverse vaginal microbiota in cynomolgus macaques makes it a good model to study dysbiosis: the NHP model can be used to test various strategies aiming at improving the genital health by modifying the vaginal microbiota for instance using combinations of antibiotics, prebiotic and/or probiotics (Figure 4).

Overall, the NHP model is suitable to study human physiological and pathological situations. It allows the monitoring of the systemic and local immune responses in the FRT. Various factors impacting the local inflammation such as cytokine profiles, immune cell distribution, the vaginal microbiota, ongoing STI … can also be evaluated individually in this model. It is a relevant model to study the susceptibility and the response to infectious diseases such as STI. Moreover, the NHP model can be manipulated to evaluated safety/efficacy of vaccines and therapies against STI or genital inflammation.

## 5. Conclusion

Vaginal microbiota composition is increasingly described to have a central role in the regulation of FRT inflammation and protection against STI acquisition. Indeed, *Lactobacillus* spp. allow the maintenance of a low inflammatory environment and are able to control STI through different mechanisms (lactic acid/bacteriocin production, adhesion to cells/pathogens etc.). Neutrophils are key modulators of the inflammation. In the FRT, their interaction with the microbiota impacts the immune response to STI. However, the mechanisms of action are still poorly understood. New models, including cervicovaginal 3D model or human vagina on chip, will be interesting to study how the vaginal microbiota composition, as well as metabolites, regulate the FRT inflammation (42, 192). However, the use of a suitable animal model that reproduce the FRT environment, including immune cells, soluble factors, vaginal microbiota composition with an environment subject to hormonal fluctuation for instance, is mandatory. Since strains specificity was observed in terms of inflammation and metabolites production (193), deep analysis at the strain level will also be essential to better understand the role of key bacteria in inflammation.

In conclusion, this review highlights the interplay between the vaginal microbiota composition and the local inflammation, including cytokine/chemokine production, cell recruitment and especially neutrophil presence in association with susceptibility to STI. Moreover, several mechanisms of action of how an optimal vaginal microbiota is able to decrease the susceptibility and favor the treatment of STI, are discussed. Finally, the NHP model is described demonstrating its usefulness for the study of vaginal microbiota composition and FRT inflammation, including innate and adaptive immune responses in a context or not of STIs.

## References

[1] Iijima N Thompson J Iwasaki A. Dendritic cells and macrophages in the genitourinary tract. Mucosal Immunol. (2008) 1:451–9. 10.1038/mi.2008.57 19079212 PMC2684461

[2] Petrova MI van den Broek M Balzarini J Vanderleyden J Lebeer S. Vaginal microbiota and its role in HIV transmission and infection. FEMS Microbiol Rev. (2013) 37:762–92. 10.1111/1574-6976.12029 23789590

[3] Ramanathan R Woodrow K. Engineering immunity in the mucosal niche against sexually transmitted infections. Wiley Interdiscip Rev Nanomed Nanobiotechnol. (2016) 8:107–22. 10.1002/wnan.1359 26153141 PMC6467227

[4] Hickey DK Patel MV Fahey JV Wira CR. Innate and adaptive immunity at mucosal surfaces of the female reproductive tract: stratification and integration of immune protection against the transmission of sexually transmitted infections. J Reprod Immunol. (2011) 88:185–94. 10.1016/j.jri.2011.01.005 21353708 PMC3094911

[5] Kaushic C. HIV-1 infection in the female reproductive tract: role of interactions between HIV-1 and genital epithelial cells. Am J Reprod Immunol. (2011) 65:253–60. 10.1111/j.1600-0897.2010.00965.x 21223427

[6] Andersch-Björkman Y Thomsson KA Larsson JMH Ekerhovd E Hansson GC. Large scale identification of proteins, mucins, and their O-glycosylation in the endocervical mucus during the menstrual cycle. Mol Cell Proteomics. (2007) 6:708–16. 10.1074/mcp.M600439-MCP200 17220477

[7] Reis Machado J da Silva MV Cavellani CL dos Reis MA Monteiro ML Teixeira Vde P Mucosal immunity in the female genital tract, HIV/AIDS. Biomed Res Int. (2014) 350195. 10.1155/2014/350195 PMC4181941 25313360

[8] Strbo N Alcaide ML Romero L Bolivar H Jones D Podack ER Loss of intraepithelial endocervical gamma delta (GD) 1 T cells in HIV infected women. Am J Reprod Immunol. (2016) 75:134–45. 10.1111/aji.12458 26666220 PMC4715976

[9] Gibbs A Leeansyah E Introini A Paquin-Proulx D Hasselrot K Andersson E MAIT cells reside in the female genital mucosa and are biased towards IL-17 and IL-22 production in response to bacterial stimulation. Mucosal Immunol. (2017) 10:35–45. 10.1038/mi.2016.30 27049062 PMC5053908

[10] Benjelloun F Quillay H Cannou C Marlin R Madec Y Fernandez H Activation of toll-like receptors differentially modulates inflammation in the human reproductive tract: preliminary findings. Front Immunol. (2020) 11:1655. 10.3389/fimmu.2020.01655 32849571 PMC7417306

[11] Trifonova RT Lieberman J van Baarle D. Distribution of immune cells in the human cervix and implications for HIV transmission. Am J Reprod Immunol. (2014) 71:252–64. 10.1111/aji.12198 24410939 PMC3943534

[12] Pudney J Quayle AJ Anderson DJ. Immunological microenvironments in the human vagina and cervix: mediators of cellular immunity are concentrated in the cervical transformation zone. Biol Reprod. (2005) 73:1253–63. 10.1095/biolreprod.105.043133 16093359

[13] Sullivan DA Richardson GS MacLaughlin DT Wira CR. Variations in the levels of secretory component in human uterine fluid during the menstrual cycle. J Steroid Biochem. (1984) 20:509–13. 10.1016/0022-4731(84)90263-2 6708533

[14] Wira CR Rodriguez-Garcia M Patel MV. The role of sex hormones in immune protection of the female reproductive tract. Nat Rev Immunol. (2015) 15:217–30. 10.1038/nri3819 25743222 PMC4716657

[15] Ravel J Gajer P Abdo Z Schneider GM Koenig SSK McCulle SL Vaginal microbiome of reproductive-age women. Proc Natl Acad Sci USA. (2011) 108(Suppl 1):4680–7. 10.1073/pnas.1002611107 20534435 PMC3063603

[16] Gajer P Brotman RM Bai G Sakamoto J Schütte UME Zhong X Temporal dynamics of the human vaginal microbiota. Sci Transl Med. (2012) 4:132ra52. 10.1126/scitranslmed.3003605 22553250 PMC3722878

[17] Anahtar MN Byrne EH Doherty KE Bowman BA Yamamoto HS Soumillon M Cervicovaginal bacteria are a major modulator of host inflammatory responses in the female genital tract. Immunity. (2015) 42:965–76. 10.1016/j.immuni.2015.04.019 25992865 PMC4461369

[18] Kumar M Murugesan S Singh P Saadaoui M Elhag DA Terranegra A Vaginal microbiota and cytokine levels predict preterm delivery in Asian women. Front Cell Infect Microbiol. (2021) 11:639665. 10.3389/fcimb.2021.639665 33747983 PMC7969986

[19] Srinivasan S Morgan MT Fiedler TL Djukovic D Hoffman NG Raftery D Metabolic signatures of bacterial vaginosis. mBio. (2015) 6(2):e00204–15. 10.1128/mBio.00204-15 25873373 PMC4453549

[20] Fredricks DN Fiedler TL Marrazzo JM. Molecular identification of bacteria associated with bacterial vaginosis. N Engl J Med. (2005) 353:1899–911. 10.1056/NEJMoa043802 16267321

[21] Criswell BS Ladwig CL Gardner HL Dukes CD. Haemophilus vaginalis: vaginitis by inoculation from culture. Obstet Gynecol. (1969) 33:195–9. 4886951

[22] Muzny CA Łaniewski P Schwebke JR Herbst-Kralovetz MM. Host-vaginal microbiota interactions in the pathogenesis of bacterial vaginosis. Curr Opin Infect Dis. (2020) 33:59–65. 10.1097/QCO.0000000000000620 31789672 PMC7265982

[23] Mirmonsef P Hotton AL Gilbert D Gioia CJ Maric D Hope TJ Glycogen levels in undiluted genital fluid and their relationship to vaginal pH, estrogen, and progesterone. PLoS One. (2016) 11:e0153553. 10.1371/journal.pone.0153553 27093050 PMC4836725

[24] Brooks JP Edwards DJ Blithe DL Fettweis JM Serrano MG Sheth NU Effects of combined oral contraceptives, depot medroxyprogesterone acetate, and the levonorgestrel-releasing intrauterine system on the vaginal microbiome. Contraception. (2017) 95:405–13. 10.1016/j.contraception.2016.11.006 27913230 PMC5376524

[25] Balle C Konstantinus IN Jaumdally SZ Havyarimana E Lennard K Esra R Hormonal contraception alters vaginal microbiota and cytokines in South African adolescents in a randomized trial. Nat Commun. (2020) 11(1):5578. 10.1038/s41467-020-19382-9 33149114 PMC7643181

[26] Schwebke JR. New concepts in the etiology of bacterial vaginosis. Curr Infect Dis Rep. (2009) 11:143–7. 10.1007/s11908-009-0021-7 19239805

[27] Alcaide ML Rodriguez VJ Brown MR Pallikkuth S Arheart K Martinez O High levels of inflammatory cytokines in the reproductive tract of women with BV and engaging in intravaginal douching: a cross-sectional study of participants in the women interagency HIV study. AIDS Res Hum Retroviruses. (2017) 33:309–17. 10.1089/aid.2016.0187 27897054 PMC5372759

[28] Mehta SD Agingu W Nordgren RK Green SJ Bhaumik DK Bailey RC Characteristics of women and their male sex partners predict bacterial vaginosis among a prospective cohort of Kenyan women with nonoptimal vaginal Microbiota. Sex Transm Dis. (2020) 47:840–50. 10.1097/OLQ.0000000000001259 32773610 PMC7668344

[29] Wiesenfeld HC Hillier SL Krohn MA Landers DV Sweet RL. Bacterial vaginosis is a strong predictor of *Neisseria gonorrhoeae* and *Chlamydia trachomatis* infection. Clin Infect Dis. (2003) 36:663–8. 10.1086/367658 12594649

[30] Borgogna J-LC Shardell MD Yeoman CJ Ghanem KG Kadriu H Ulanov AV The association of *Chlamydia trachomatis* and *Mycoplasma genitalium* infection with the vaginal metabolome. Sci Rep. (2020) 10(1):3420. 10.1038/s41598-020-60179-z 32098988 PMC7042340

[31] Kroon SJ Ravel J Huston WM. Cervicovaginal microbiota, women’s health, and reproductive outcomes. Fertil Steril. (2018) 110:327–36. 10.1016/j.fertnstert.2018.06.036 30098679

[32] Carda-Diéguez M Cárdenas N Aparicio M Beltrán D Rodríguez JM Mira A. Variations in vaginal, penile, and oral microbiota after sexual intercourse: a case report. Front Med. (2019) 6:178. 10.3389/fmed.2019.00178 PMC6692966 31440511

[33] Balle C Esra R Havyarimana E Jaumdally SZ Lennard K Konstantinus IN Relationship between the oral and vaginal microbiota of South African adolescents with high prevalence of bacterial vaginosis. Microorganisms. (2020) 8(7):1004. 10.3390/microorganisms8071004 32635588 PMC7409319

[34] Song SD Acharya KD Zhu JE Deveney CM Walther-Antonio MRS Tetel MJ Daily vaginal microbiota fluctuations associated with natural hormonal cycle, contraceptives, *diet, and exercise*. mSphere. (2020) 5(4):e00593-20. 10.1128/mSphere.00593-20 32641429 PMC7343982

[35] Brotman RM He X Gajer P Fadrosh D Sharma E Mongodin EF Association between cigarette smoking and the vaginal microbiota: a pilot study. BMC Infect Dis. (2014) 14:471. 10.1186/1471-2334-14-471 25169082 PMC4161850

[36] Amabebe E Anumba DOC. The vaginal microenvironment: the physiologic role of lactobacilli. Front Med. (2018) 5:181. 10.3389/fmed.2018.00181 PMC6008313 29951482

[37] Lennard K Dabee S Barnabas SL Havyarimana E Blakney A Jaumdally SZ Microbial composition predicts genital tract inflammation and persistent bacterial vaginosis in South African adolescent females. Infect Immun. (2018) 86(1):e00410–17. 10.1128/IAI.00410-17 29038128 PMC5736802

[38] Masson L Barnabas S Deese J Lennard K Dabee S Gamieldien H Inflammatory cytokine biomarkers of asymptomatic sexually transmitted infections and vaginal dysbiosis: a multicentre validation study. Sex Transm Infect. (2019) 95:5–12. 10.1136/sextrans-2017-053506 30018088

[39] Jespers V Kyongo J Joseph S Hardy L Cools P Crucitti T A longitudinal analysis of the vaginal microbiota and vaginal immune mediators in women from sub-saharan Africa. Sci Rep. (2017) 7:11974. 10.1038/s41598-017-12198-6 28931859 PMC5607244

[40] Gautam R Borgdorff H Jespers V Francis SC Verhelst R Mwaura M Correlates of the molecular vaginal microbiota composition of african women. BMC Infect Dis. (2015) 15:86. 10.1186/s12879-015-0831-1 25887567 PMC4343073

[41] Eade CR Diaz C Wood MP Anastos K Patterson BK Gupta P Identification and characterization of bacterial vaginosis-associated pathogens using a comprehensive cervical-vaginal epithelial coculture assay. Plos One. (2012) 7(11):e50106. 10.1371/journal.pone.0050106 23166828 PMC3499514

[42] Łaniewski P Herbst-Kralovetz MM. Bacterial vaginosis and health-associated bacteria modulate the immunometabolic landscape in 3D model of human cervix. NPJ Biofilms Microbiomes. (2021) 7:88. 10.1038/s41522-021-00259-8 34903740 PMC8669023

[43] Borgdorff H Gautam R Armstrong SD Xia D Ndayisaba GF van Teijlingen NH Cervicovaginal microbiome dysbiosis is associated with proteome changes related to alterations of the cervicovaginal mucosal barrier. Mucosal Immunol. (2016) 9:621–33. 10.1038/mi.2015.86 26349657

[44] Delgado-Diaz DJ Tyssen D Hayward JA Gugasyan R Hearps AC Tachedjian G. Distinct immune responses elicited from cervicovaginal epithelial cells by lactic acid and short chain fatty acids associated with optimal and non-optimal vaginal microbiota. Front Cell Infect Microbiol. (2020) 9:446. 10.3389/fcimb.2019.00446 31998660 PMC6965070

[45] Jang S-E Jeong J-J Choi S-Y Kim H Han MJ Kim D-H. Lactobacillus rhamnosus HN001 and *Lactobacillus* acidophilus la-14 attenuate *Gardnerella vaginalis*-infected bacterial vaginosis in mice. Nutrients. (2017) 9(6):531. 10.3390/nu9060531 28545241 PMC5490510

[46] Nicolò S Tanturli M Mattiuz G Antonelli A Baccani I Bonaiuto C Vaginal lactobacilli and vaginal dysbiosis-associated bacteria differently affect cervical epithelial and immune homeostasis and anti-viral defenses. IJMS. (2021) 22:6487. 10.3390/ijms22126487 34204294 PMC8234132

[47] Gosmann C Anahtar MN Handley SA Farcasanu M Abu-Ali G Bowman BA Lactobacillus-deficient cervicovaginal bacterial communities are associated with increased HIV acquisition in young South African women. Immunity. (2017) 46:29–37. 10.1016/j.immuni.2016.12.013 28087240 PMC5270628

[48] Ssemaganda A Cholette F Perner M Kambaran C Adhiambo W Wambugu PM Endocervical regulatory T cells are associated with decreased genital inflammation and lower HIV target cell abundance. Front Immunol. (2021) 12:726472. 10.3389/fimmu.2021.726472 34630402 PMC8495419

[49] Munusamy Ponnan S Thiruvengadam K Tellapragada C Ambikan AT Narayanan A Kathirvel S Deciphering the role of mucosal immune responses and the cervicovaginal microbiome in resistance to HIV infection in HIV-exposed seronegative (HESN) women. Microbiol Spectr. (2021) 9:e00470–21. 10.1128/Spectrum.00470-21 34704803 PMC8549735

[50] Byrne EH Farcasanu M Bloom SM Xulu N Xu J Hykes BL Antigen presenting cells link the female genital tract microbiome to mucosal inflammation, with hormonal contraception as an additional modulator of inflammatory signatures. Front Cell Infect Microbiol. (2021) 11:882. 10.3389/fcimb.2021.733619 PMC8482842 34604114

[51] Thurman AR Kimble T Herold B Mesquita PMM Fichorova RN Dawood HY Bacterial vaginosis and subclinical markers of genital tract inflammation and mucosal immunity. AIDS Res Hum Retroviruses. (2015) 31:1139–52. 10.1089/aid.2015.0006 26204200 PMC4651020

[52] Wira CR Fahey JV Sentman CL Pioli PA Shen L. Innate and adaptive immunity in female genital tract: cellular responses and interactions. Immunol Rev. (2005) 206:306–35. 10.1111/j.0105-2896.2005.00287.x 16048557

[53] Smith JM Wira CR Fanger MW Shen L. Human fallopian tube neutrophils – A distinct phenotype from blood neutrophils. Am J Reprod Immunol. (2006) 56:218–29. 10.1111/j.1600-0897.2006.00410.x 16938110

[54] Lasarte S Samaniego R Salinas-Muñoz L Guia-Gonzalez MA Weiss LA Mercader E Sex hormones coordinate neutrophil immunity in the vagina by controlling chemokine gradients. J Infect Dis. (2016) 213:476–84. 10.1093/infdis/jiv402 26238687

[55] Godaly G Proudfoot AE Offord RE Svanborg C Agace WW. Role of epithelial interleukin-8 (IL-8) and neutrophil IL-8 receptor A in *Escherichia coli*-induced transuroepithelial neutrophil migration. Infect Immun. (1997) 65:3451–6. 10.1128/iai.65.8.3451-3456.1997 9234811 PMC175488

[56] Sasaki S Nagata K Kobayashi Y. Regulation of the estrous cycle by neutrophil infiltration into the vagina. Biochem Biophys Res Commun. (2009) 382:35–40. 10.1016/j.bbrc.2009.02.112 19249292

[57] Salinas-Muñoz L Campos-Fernández R Mercader E Olivera-Valle I Fernández-Pacheco C Matilla L Estrogen receptor-alpha (ESR1) governs the lower female reproductive tract vulnerability to *Candida albicans*. Front Immunol. (2018) 9:1033. 10.3389/fimmu.2018.01033 29881378 PMC5976782

[58] Adapen C Réot L Nunez N Cannou C Marlin R Lemaître J Local innate markers and vaginal microbiota composition are influenced by hormonal cycle phases. Front Immunol. (2022) 13:841723. 10.3389/fimmu.2022.841723 35401577 PMC8990777

[59] Zhang D Frenette PS. Cross talk between neutrophils and the microbiota. Blood. (2019) 133:2168–77. 10.1182/blood-2018-11-844555 30898860 PMC6524562

[60] Mohammadi A Bagherichimeh S Perry MC Fazel A Tevlin E Huibner S The impact of cervical cytobrush sampling on cervico-vaginal immune parameters and microbiota relevant to HIV susceptibility. Sci Rep. (2020) 10(1):8514. 10.1038/s41598-020-65544-6 32444843 PMC7244754

[61] Cauci S. Interrelationships of interleukin-8 with interleukin-1beta and neutrophils in vaginal fluid of healthy and bacterial vaginosis positive women. Mol Hum Reprod. (2003) 9:53–8. 10.1093/molehr/gag003 12529421

[62] Chen Q Wang S Guo J Xie Q Evivie SE Song Y The protective effects of *Lactobacillus* plantarum KLDS 1.0344 on LPS-induced mastitis in vitro and in vivo. Front Immunol. (2021) 12:770822. 10.3389/fimmu.2021.770822 34858427 PMC8630701

[63] Michels M Jesus GFA Voytena APL Rossetto M Ramlov F Córneo E Immunomodulatory effect of *Bifidobacterium*, *Lactobacillus*, and *Streptococcus* strains of paraprobiotics in lipopolysaccharide-stimulated inflammatory responses in RAW-264.7 macrophages. Curr Microbiol. (2022) 79:9. 10.1007/s00284-021-02708-1 34905100

[64] Molloy MJ Grainger JR Bouladoux N Hand TW Koo LY Naik S Intraluminal containment of commensal outgrowth in the gut during infection-induced dysbiosis. Cell Host Microbe. (2013) 14:318–28. 10.1016/j.chom.2013.08.003 24034617 PMC4806337

[65] Vogt KL Summers C Chilvers ER Condliffe AM. Priming and de-priming of neutrophil responses in vitro and in vivo. Eur J Clin Invest. (2018) 48:e12967. 10.1111/eci.12967 29896919

[66] Cassatella MA Östberg NK Tamassia N Soehnlein O. Biological roles of neutrophil-derived granule proteins and cytokines. Trends Immunol. (2019) 40:648–64. 10.1016/j.it.2019.05.003 31155315

[67] Yoshimura T McLean MH Dzutsev AK Yao X Chen K Huang J The antimicrobial peptide CRAMP is essential for colon homeostasis by maintaining microbiota balance. J Immunol. (2018) 200:2174–85. 10.4049/jimmunol.1602073 29440355 PMC5931736

[68] Barr FD Ochsenbauer C Wira CR Rodriguez-Garcia M. Neutrophil extracellular traps prevent HIV infection in the female genital tract. Mucosal Immunol. (2018) 11:1420–8. 10.1038/s41385-018-0045-0 29875403 PMC6162173

[69] Winterbourn CC Kettle AJ Hampton MB. Reactive oxygen species and neutrophil function. Annu Rev Biochem. (2016) 85:765–92. 10.1146/annurev-biochem-060815-014442 27050287

[70] Hahn S Giaglis S Chowdury CS Hösli I Hasler P. Modulation of neutrophil NETosis: interplay between infectious agents and underlying host physiology. Semin Immunopathol. (2013) 35:439–53. 10.1007/s00281-013-0380-x 23649713 PMC3685704

[71] Bornhöfft KF Rebl A Gallagher ME Viergutz T Zlatina K Reid C Sialylated cervical mucins inhibit the activation of neutrophils to form neutrophil extracellular traps in bovine in vitro model. Front Immunol. (2019) 10:2478. 10.3389/fimmu.2019.02478 31781090 PMC6851059

[72] Lewis WG Robinson LS Gilbert NM Perry JC Lewis AL. Degradation, foraging, and depletion of mucus sialoglycans by the vagina-adapted actinobacterium *Gardnerella vaginalis*. J Biol Chem. (2013) 288:12067–79. 10.1074/jbc.M113.453654 23479734 PMC3636892

[73] Deshmukh HS Liu Y Menkiti OR Mei J Dai N O’Leary CE The microbiota regulates neutrophil homeostasis and host resistance to *Escherichia coli* K1 sepsis in neonatal mice. Nat Med. (2014) 20:524–30. 10.1038/nm.3542 24747744 PMC4016187

[74] Schluter J Peled JU Taylor BP Markey KA Smith M Taur Y The gut microbiota is associated with immune cell dynamics in humans. Nature. (2020) 588:303–7. 10.1038/s41586-020-2971-8 33239790 PMC7725892

[75] Mtshali A San JE Osman F Garrett N Balle C Giandhari J Temporal changes in vaginal microbiota and genital tract cytokines among South African women treated for bacterial vaginosis. Front Immunol. (2021) 12:730986. 10.3389/fimmu.2021.730986 34594336 PMC8477043

[76] Fagundes CT Amaral FA Vieira AT Soares AC Pinho V Nicoli JR Transient TLR activation restores inflammatory response and ability to control pulmonary bacterial infection in germfree mice. J Immunol. (2012) 188:1411–20. 10.4049/jimmunol.1101682 22210917

[77] Zhang D Chen G Manwani D Mortha A Xu C Faith JJ Neutrophil ageing is regulated by the microbiome. Nature. (2015) 525:528–32. 10.1038/nature15367 26374999 PMC4712631

[78] Wang Y Gu Y Fang K Mao K Dou J Fan H *Lactobacillus acidophilus* and *Clostridium butyricum* ameliorate colitis in murine by strengthening the gut barrier function and decreasing inflammatory factors. Benef Microbes. (2018) 9:775–87. 10.3920/BM2017.0035 30014710

[79] Shi C Jia T Mendez-Ferrer S Hohl TM Serbina NV Lipuma L Bone marrow mesenchymal stem and progenitor cells induce monocyte emigration in response to circulating toll-like receptor ligands. Immunity. (2011) 34:590–601. 10.1016/j.immuni.2011.02.016 21458307 PMC3081416

[80] Vinolo MAR Rodrigues HG Hatanaka E Hebeda CB Farsky SHP Curi R. Short-chain fatty acids stimulate the migration of neutrophils to inflammatory sites. Clin Sci. (2009) 117:331–8. 10.1042/CS20080642 19335337

[81] Ohkubo T Tsuda M Tamura M Yamamura M. Impaired superoxide production in peripheral blood neutrophils of germ-free rats. Scand J Immunol. (1990) 32:727–9. 10.1111/j.1365-3083.1990.tb03216.x 1702900

[82] Clarke TB Davis KM Lysenko ES Zhou AY Yu Y Weiser JN. Recognition of peptidoglycan from the microbiota by Nod1 enhances systemic innate immunity. Nat Med. (2010) 16:228–31. 10.1038/nm.2087 20081863 PMC4497535

[83] Li G Lin J Zhang C Gao H Lu H Gao X Microbiota metabolite butyrate constrains neutrophil functions and ameliorates mucosal inflammation in inflammatory bowel disease. Gut Microbes. (2021) 13:1968257. 10.1080/19490976.2021.1968257 34494943 PMC8437544

[84] Vinolo MAR Rodrigues HG Hatanaka E Sato FT Sampaio SC Curi R. Suppressive effect of short-chain fatty acids on production of proinflammatory mediators by neutrophils. J Nutr Biochem. (2011) 22:849–55. 10.1016/j.jnutbio.2010.07.009 21167700

[85] Martin HL Richardson BA Nyange PM Lavreys L Hillier SL Chohan B Vaginal lactobacilli, microbial flora, and risk of human immunodeficiency virus type 1 and sexually transmitted disease acquisition. J Infect Dis. (1999) 180:1863–8. 10.1086/315127 10558942

[86] Sewankambo N Gray RH Wawer MJ Paxton L McNairn D Wabwire-Mangen F HIV-1 infection associated with abnormal vaginal flora morphology and bacterial vaginosis. Lancet. (1997) 350:546–50. 10.1016/S0140-6736(97)01063-5 9284776

[87] Brotman RM Klebanoff MA Nansel TR Yu KF Andrews WW Zhang J Bacterial vaginosis assessed by gram stain and diminished colonization resistance to incident gonococcal, chlamydial, and trichomonal genital infection. J Infect Dis. (2010) 202:1907–15. 10.1086/657320 21067371 PMC3053135

[88] Balle C Lennard K Dabee S Barnabas SL Jaumdally SZ Gasper MA Endocervical and vaginal microbiota in South African adolescents with asymptomatic *Chlamydia trachomatis* infection. Sci Rep. (2018) 8(1):11109. 10.1038/s41598-018-29320-x 30038262 PMC6056523

[89] Ceccarani C Foschi C Parolin C D’Antuono A Gaspari V Consolandi C Diversity of vaginal microbiome and metabolome during genital infections. Sci Rep. (2019) 9:14095. 10.1038/s41598-019-50410-x 31575935 PMC6773718

[90] Lovett A Seña AC Macintyre AN Sempowski GD Duncan JA Waltmann A. Cervicovaginal microbiota predicts *Neisseria gonorrhoeae* clinical presentation. Front Microbiol. (2022) 12:790531. 10.3389/fmicb.2021.790531 35222300 PMC8867028

[91] Chen H Wang L Zhao L Luo L Min S Wen Y Alterations of vaginal microbiota in women with infertility and *Chlamydia trachomatis* infection. Front Cell Infect Microbiol. (2021) 11:698840. 10.3389/fcimb.2021.698840 34414130 PMC8370387

[92] Chiu S-F Huang P-J Cheng W-H Huang C-Y Chu LJ Lee C-C Vaginal microbiota of the sexually transmitted infections caused by *Chlamydia trachomatis* and trichomonas vaginalis in women with vaginitis in Taiwan. Microorganisms. (2021) 9:1864. 10.3390/microorganisms9091864 34576759 PMC8470505

[93] Jarrett OD Srinivasan S Richardson BA Fiedler T Wallis JM Kinuthia J Specific vaginal bacteria are associated with an increased risk of trichomonas vaginalis acquisition in women. J Infect Dis. (2019) 220:1503–10. 10.1093/infdis/jiz354 31287879 PMC6761949

[94] Raimondi S Candeliere F Amaretti A Foschi C Morselli S Gaspari V Vaginal and anal microbiome during *Chlamydia trachomatis* infections. Pathogens. (2021) 10:1347. 10.3390/pathogens10101347 34684295 PMC8539191

[95] Taylor-Robinson D. Mollicutes in vaginal microbiology: mycoplasma hominis, *Ureaplasma urealyticum*, *Ureaplasma parvum* and *Mycoplasma genitalium*. Res Microbiol. (2017) 168:875–81. 10.1016/j.resmic.2017.02.009 28263902

[96] Jonduo ME Vallely LM Wand H Sweeney EL Egli-Gany D Kaldor J Adverse pregnancy and birth outcomes associated with *Mycoplasma hominis*, *Ureaplasma urealyticum* and *Ureaplasma parvum*: a systematic review and meta-analysis. BMJ Open. (2022) 12:e062990. 10.1136/bmjopen-2022-062990 36028274 PMC9422885

[97] Miyoshi Y Suga S Sugimi S Kurata N Yamashita H Yasuhi I. Vaginal *Ureaplasma urealyticum* or *Mycoplasma hominis* and preterm delivery in women with threatened preterm labor. J Matern Fetal Neonatal Med. (2022) 35:878–83. 10.1080/14767058.2020.1733517 32131651

[98] Wei Z-T Chen H-L Wang C-F Yang G-L Han S-M Zhang S-L. Depiction of vaginal microbiota in women with high-risk human papillomavirus infection. Front Public Health. (2021) 8:587298. 10.3389/fpubh.2020.587298 33490017 PMC7820762

[99] Borgogna J Shardell M Santori E Nelson T Rath J Glover E The vaginal metabolome and microbiota of cervical HPV-positive and HPV-negative women: a cross-sectional analysis. BJOG: Int J Obstet Gy. (2020) 127:182–92. 10.1111/1471-0528.15981 PMC6982399 31749298

[100] Tamarelle J Thiébaut ACM de Barbeyrac B Bébéar C Ravel J Delarocque-Astagneau E. The vaginal microbiota and its association with human papillomavirus, *Chlamydia trachomatis*, *Neisseria gonorrhoeae* and *Mycoplasma genitalium* infections: a systematic review and meta-analysis. Clin Microbiol Infect. (2019) 25:35–47. 10.1016/j.cmi.2018.04.019 29729331 PMC7362580

[101] Lebeau A Bruyere D Roncarati P Peixoto P Hervouet E Cobraiville G HPV Infection alters vaginal microbiome through down-regulating host mucosal innate peptides used by lactobacilli as amino acid sources. Nat Commun. (2022) 13:1076. 10.1038/s41467-022-28724-8 35228537 PMC8885657

[102] Caselli E D’Accolti M Santi E Soffritti I Conzadori S Mazzacane S Vaginal microbiota and cytokine microenvironment in HPV clearance/persistence in women surgically treated for cervical intraepithelial neoplasia: an observational prospective study. Front Cell Infect Microbiol. (2020) 10:540900. 10.3389/fcimb.2020.540900 33251154 PMC7676899

[103] Moscicki A-B Shi B Huang H Barnard E Li H. Cervical-vaginal microbiome and associated cytokine profiles in a prospective study of HPV 16 acquisition, persistence, and clearance. Front Cell Infect Microbiol. (2020) 10:569022. 10.3389/fcimb.2020.569022 33102255 PMC7546785

[104] McClelland RS Lingappa JR Srinivasan S Kinuthia J John-Stewart GC Jaoko W Evaluation of the association between the concentrations of key vaginal bacteria and the increased risk of HIV acquisition in african women from five cohorts: a nested case-control study. Lancet Infect Dis. (2018) 18:554–64. 10.1016/S1473-3099(18)30058-6 29396006 PMC6445552

[105] Pyles RB Vincent KL Baum MM Elsom B Miller AL Maxwell C Cultivated vaginal microbiomes Alter HIV-1 infection and antiretroviral efficacy in colonized epithelial multilayer cultures. PLoS One. (2014) 9(3):e93419. 10.1371/journal.pone.0093419 24676219 PMC3968159

[106] Ghys PD Fransen K Diallo MO Ettiègne-Traoré V Coulibaly IM Yeboué KM The associations between cervicovaginal HIV shedding, sexually transmitted diseases and immunosuppression in female sex workers in abidjan, côte d’Ivoire. AIDS. (1997) 11:F85–93. 10.1097/00002030-199712000-00001 9342059

[107] Venkatesh KK van der Straten A Cheng H Montgomery ET Lurie MN Chipato T The relative contribution of viral and bacterial sexually transmitted infections on HIV acquisition in southern african women in the methods for improving reproductive health in Africa study. Int J STD AIDS. (2011) 22:218–24. 10.1258/ijsa.2010.010385 21515755

[108] Peterman TA Newman DR Maddox L Schmitt K Shiver S. Risk for HIV following a diagnosis of syphilis, gonorrhoea or chlamydia: 328,456 women in Florida, 2000–2011. Int J STD AIDS. (2015) 26:113–9. 10.1177/0956462414531243 24713228 PMC6755665

[109] Sperling R Kraus TA Ding J Veretennikova A Lorde-Rollins E Singh T Differential profiles of immune mediators and in vitro HIV infectivity between endocervical and vaginal secretions from women with *Chlamydia trachomatis* infection: a pilot study. J Reprod Immunol. (2013) 99(1-2):80–7. 10.1016/j.jri.2013.07.003 23993451 PMC3874462

[110] Schust DJ Ibana JA Buckner LR Ficarra M Sugimoto J Amedee AM Potential mechanisms for increased HIV-1 transmission across the endocervical epithelium during C. trachomatis infection. Curr HIV Res. (2012) 10:218–27. 10.2174/157016212800618093 22384841 PMC3870887

[111] Kelly KA Walker JC Jameel SH Gray HL Rank RG. Differential regulation of CD4 lymphocyte recruitment between the upper and lower regions of the genital tract during Chlamydia trachomatis infection. Infect Immun. (2000) 68:1519–28. 10.1128/IAI.68.3.1519-1528.2000 10678969 PMC97310

[112] Keller MJ Huber A Espinoza L Serrano MG Parikh HI Buck GA Impact of herpes simplex virus type 2 and human immunodeficiency virus dual infection on female genital tract mucosal immunity and the vaginal microbiome. J Infect Dis. (2019) 220:852–61. 10.1093/infdis/jiz203 31111902 PMC6667798

[113] Di Pietro M Filardo S Porpora MG Recine N Latino MA Sessa R. HPV/*Chlamydia trachomatis* co-infection: metagenomic analysis of cervical microbiota in asymptomatic women. New Microbiologica. (2018) 41(1):34–41. 29313867

[114] Hensley-McBain T Wu MC Manuzak JA Cheu RK Gustin A Driscoll CB Increased mucosal neutrophil survival is associated with altered microbiota in HIV infection. PLoS Pathog. (2019) 15:e1007672. 10.1371/journal.ppat.1007672 30973942 PMC6459500

[115] Hernandez JC Giraldo DM Paul S Urcuqui-Inchima S. Involvement of neutrophil hyporesponse and the role of toll-like receptors in human immunodeficiency virus 1 protection. Plos One. (2015) 10:e0119844. 10.1371/journal.pone.0119844 25785697 PMC4364960

[116] Vujkovic-Cvijin I Sortino O Verheij E Sklar J Wit FW Kootstra NA HIV-associated gut dysbiosis is independent of sexual practice and correlates with noncommunicable diseases. Nat Commun. (2020) 11:2448. 10.1038/s41467-020-16222-8 32415070 PMC7228978

[117] Hensley-McBain T Klatt NR. The dual role of neutrophils in HIV infection. Curr HIV/AIDS Rep. (2018) 15:1–10. 10.1007/s11904-018-0370-7 29516266 PMC6086572

[118] Lehr S Vier J Häcker G Kirschnek S. Activation of neutrophils by *Chlamydia trachomatis*-infected epithelial cells is modulated by the chlamydial plasmid. Microbes Infect. (2018) 20:284–92. 10.1016/j.micinf.2018.02.007 29499390

[119] Jean S Juneau RA Criss AK Cornelissen CN. Neisseria gonorrhoeae evades calprotectin-mediated nutritional immunity and survives neutrophil extracellular traps by production of TdfH. Infect Immun. (2016) 84:2982–94. 10.1128/IAI.00319-16 27481245 PMC5038063

[120] Rajeeve K Das S Prusty BK Rudel T. Chlamydia trachomatis paralyses neutrophils to evade the host innate immune response. Nat Microbiol. (2018) 3:824. 10.1038/s41564-018-0182-y 29946164

[121] Handing JW Criss AK. The lipooligosaccharide-modifying enzyme LptA enhances gonococcal defence against human neutrophils: LptA and gonococcal defence from neutrophils. Cell Microbiol. (2015) 17:910–21. 10.1111/cmi.12411 25537831 PMC4437829

[122] Vareille-Delarbre M Miquel S Garcin S Bertran T Balestrino D Evrard B Immunomodulatory effects of Lactobacillus plantarum on inflammatory response induced by *Klebsiella pneumoniae*. Infect Immun. (2019) 87(11):e00570–19. 10.1128/IAI.00570-19 31481408 PMC6803346

[123] Martín R Soberón N Vaneechoutte M Flórez AB Vázquez F Suárez JE. Characterization of indigenous vaginal lactobacilli from healthy women as probiotic candidates. Int Microbiol. (2008) 11:261–6. 10.2436/20.1501.01.70 19204898

[124] Martín R Sánchez B Suárez JE Urdaci MC. Characterization of the adherence properties of human lactobacilli strains to be used as vaginal probiotics. FEMS Microbiol Lett. (2012) 328:166–73. 10.1111/j.1574-6968.2011.02495.x 22224921

[125] Mastromarino P Di Pietro M Schiavoni G Nardis C Gentile M Sessa R. Effects of vaginal lactobacilli in *Chlamydia trachomatis* infection. Int J Med Microbiol. (2014) 304:654–61. 10.1016/j.ijmm.2014.04.006 24875405

[126] Gong Z Luna Y Yu P Fan H. Lactobacilli inactivate *Chlamydia trachomatis* through lactic acid but not H2O2. Plos One. (2014) 9(9):e107758. 10.1371/journal.pone.0107758 25215504 PMC4162611

[127] Nardini P Ñahui Palomino RA Parolin C Laghi L Foschi C Cevenini R Lactobacillus crispatus inhibits the infectivity of *Chlamydia trachomatis* elementary bodies, in vitro study. Sci Rep. (2016) 6:29024. 10.1038/srep29024 27354249 PMC4926251

[128] Rizzo A Florentin M Buommino E Donnarumma G Losacco A Bevilacqua N. Lactobacillus crispatus mediates anti-inflammatory cytokine interleukin-10 induction in response to *Chlamydia trachomatis* infection in vitro. Int J Med Microbiol. (2015) 305(8):815–27. 10.1016/j.ijmm.2015.07.005 26372530

[129] Płaczkiewicz J Chmiel P Malinowska E Bącal P Kwiatek A. Lactobacillus crispatus and its enolase and glutamine synthetase influence interactions between *Neisseria gonorrhoeae* and human epithelial cells. J Microbiol. (2020) 58:405–14. 10.1007/s12275-020-9505-9 32279277

[130] Cheng C Zhang L Mu J Tian Q Liu Y Ma X Effect of *Lactobacillus johnsonii* strain SQ0048 on the TLRs-MyD88/NF-κB signaling pathway in bovine vaginal epithelial cells. Front Vet Sci. (2021) 8:670949. 10.3389/fvets.2021.670949 34447797 PMC8383737

[131] Liu M Wu Q Wang M Fu Y Wang J. Lactobacillus rhamnosus GR-1 limits *Escherichia coli*-induced inflammatory responses via attenuating MyD88-dependent and MyD88-independent pathway activation in bovine endometrial epithelial cells. Inflammation. (2016) 39:1483–94. 10.1007/s10753-016-0382-7 27236308

[132] Phukan N Parsamand T Brooks AES Nguyen TNM Simoes-Barbosa A. The adherence of trichomonas vaginalis to host ectocervical cells is influenced by lactobacilli. Sex Transm Infect. (2013) 89:455–9. 10.1136/sextrans-2013-051039 23720602

[133] Breedveld AC Schuster HJ van Houdt R Painter RC Mebius RE van der Veer C Enhanced IgA coating of bacteria in women with Lactobacillus crispatus-dominated vaginal microbiota. Microbiome. (2022) 10:15. 10.1186/s40168-021-01198-4 35074009 PMC8787895

[134] Ocaña VS Pesce de Ruiz Holgado AA Nader-Macías ME. Selection of vaginal H2O2-generating Lactobacillus species for probiotic use. Curr Microbiol. (1999) 38:279–84. 10.1007/pl00006802 10355116

[135] O’Hanlon DE Lanier BR Moench TR Cone RA. Cervicovaginal fluid and semen block the microbicidal activity of hydrogen peroxide produced by vaginal lactobacilli. BMC Infect Dis. (2010) 10:120. 10.1186/1471-2334-10-120 20482854 PMC2887447

[136] Witkin SS Mendes-Soares H Linhares IM Jayaram A Ledger WJ Forney LJ. Influence of vaginal bacteria and d- and l-lactic acid isomers on vaginal extracellular matrix metalloproteinase inducer: implications for protection against upper genital tract infections. mBio. (2013) 4:e00460–13. 10.1128/mBio.00460-13 23919998 PMC3735189

[137] Boskey ER Telsch KM Whaley KJ Moench TR Cone RA. Acid production by vaginal flora in vitro is consistent with the rate and extent of vaginal acidification. Infect Immun. (1999) 67:5170–5. 10.1128/IAI.67.10.5170-5175.1999 10496892 PMC96867

[138] Mirmonsef P Hotton AL Gilbert D Burgad D Landay A Weber KM Free glycogen in vaginal fluids is associated with Lactobacillus colonization and low vaginal pH. PLoS One. (2014) 9:e102467. 10.1371/journal.pone.0102467 25033265 PMC4102502

[139] Breshears LM Edwards VL Ravel J Peterson ML. Lactobacillus crispatus inhibits growth of *Gardnerella vaginalis* and *Neisseria gonorrhoeae* on a porcine vaginal mucosa model. BMC Microbiol. (2015) 15:276. 10.1186/s12866-015-0608-0 26652855 PMC4675025

[140] Tyssen D Wang Y-Y Hayward JA Agius PA DeLong K Aldunate M Anti-HIV-1 activity of lactic acid in human cervicovaginal fluid. mSphere. (2018) 3(4):e00055–18. 10.1128/mSphere.00055-18 29976641 PMC6034077

[141] Nunn KL Wang Y-Y Harit D Humphrys MS Ma B Cone R Enhanced trapping of HIV-1 by human cervicovaginal mucus is associated with *Lactobacillus crispatus*-dominant microbiota. MBio. (2015) 6:e01084–01015. 10.1128/mBio.01084-15 26443453 PMC4611035

[142] Hoang T Toler E DeLong K Mafunda NA Bloom SM Zierden HC The cervicovaginal mucus barrier to HIV-1 is diminished in bacterial vaginosis. PLoS Pathog. (2020) 16(1):e1008236. 10.1371/journal.ppat.1008236 31971984 PMC6999914

[143] Ñahui Palomino RA Zicari S Vanpouille C Vitali B Margolis L. Vaginal *Lactobacillus* inhibits HIV-1 replication in human tissues ex vivo. Front Microbiol. (2017) 8:906. 10.3389/fmicb.2017.00906 28579980 PMC5437121

[144] Aroutcheva A Gariti D Simon M Shott S Faro J Simoes JA Defense factors of vaginal lactobacilli. Am J Obstet Gynecol. (2001) 185:375–9. 10.1067/mob.2001.115867 11518895

[145] Stoyancheva G Marzotto M Dellaglio F Torriani S. Bacteriocin production and gene sequencing analysis from vaginal *Lactobacillus* strains. Arch Microbiol. (2014) 196:645–53. 10.1007/s00203-014-1003-1 24919535

[146] Cotter PD Hill C Ross RP. Bacteriocins: developing innate immunity for food. Nat Rev Microbiol. (2005) 3:777–88. 10.1038/nrmicro1273 16205711

[147] Ocaña VS Pesce de Ruiz Holgado AA Nader-Macías ME. Characterization of a bacteriocin-like substance produced by a vaginal *Lactobacillus salivarius* strain. Appl Environ Microbiol. (1999) 65:5631–5. 10.1128/AEM.65.12.5631-5635.1999 10584033 PMC91773

[148] Nilsen T Swedek I Lagenaur LA Parks TP. Novel selective inhibition of *Lactobacillus* iners by *Lactobacillus* -derived bacteriocins. Appl Environ Microbiol. (2020) 86:e01594–20. 10.1128/AEM.01594-20 32801180 PMC7531956

[149] Maldonado-Barragán A Caballero-Guerrero B Martín V Ruiz-Barba JL Rodríguez JM. Purification and genetic characterization of gassericin E, a novel co-culture inducible bacteriocin from Lactobacillus gasseri EV1461 isolated from the vagina of a healthy woman. BMC Microbiol. (2016) 16:37. 10.1186/s12866-016-0663-1 26969428 PMC4788914

[150] Gaspar C Donders GG Palmeira-de-Oliveira R Queiroz JA Tomaz C Martinez-de-Oliveira J Bacteriocin production of the probiotic *Lactobacillus acidophilus* KS400. AMB Expr. (2018) 8:153. 10.1186/s13568-018-0679-z PMC6160374 30264211

[151] Brown L Wolf JM Prados-Rosales R Casadevall A. Through the wall: extracellular vesicles in gram-positive bacteria, mycobacteria and fungi. Nat Rev Microbiol. (2015) 13:620–30. 10.1038/nrmicro3480 26324094 PMC4860279

[152] Ñahui Palomino RA Vanpouille C Laghi L Parolin C Melikov K Backlund P Extracellular vesicles from symbiotic vaginal lactobacilli inhibit HIV-1 infection of human tissues. Nat Commun. (2019) 10:5656. 10.1038/s41467-019-13468-9 31827089 PMC6906448

[153] Costantini PE Vanpouille C Firrincieli A Cappelletti M Margolis L Ñahui Palomino RA. Extracellular vesicles generated by gram-positive bacteria protect human tissues ex vivo from HIV-1 infection. Front Cell Infect Microbiol. (2022) 11:822882. 10.3389/fcimb.2021.822882 35145925 PMC8821821

[154] Ziklo N Huston WM Taing K Katouli M Timms P. In vitro rescue of genital strains of *Chlamydia trachomatis* from interferon-γ and tryptophan depletion with indole-positive, but not indole-negative *Prevotella* spp. BMC Microbiol. (2016) 16(1):286. 10.1186/s12866-016-0903-4 27914477 PMC5135834

[155] Østergaard O Follmann F Olsen AW Heegaard NH Andersen P Rosenkrands I. Quantitative protein profiling of *Chlamydia trachomatis* growth forms reveals defense strategies against tryptophan starvation. Mol Cell Proteomics. (2016) 15:3540–50. 10.1074/mcp.M116.061986 27784728 PMC5141270

[156] Caldwell HD Wood H Crane D Bailey R Jones RB Mabey D Polymorphisms in *Chlamydia trachomatis* tryptophan synthase genes differentiate between genital and ocular isolates. J Clin Invest. (2003) 111:1757–69. 10.1172/JCI200317993 12782678 PMC156111

[157] Ziklo N Vidgen ME Taing K Huston WM Timms P. Dysbiosis of the vaginal microbiota and higher vaginal kynurenine/tryptophan ratio reveals an association with *Chlamydia trachomatis* genital infections. Front Cell Infect Microbiol. (2018) 8:1. 10.3389/fcimb.2018.00001 29404279 PMC5778109

[158] Ziklo N Huston WM Hocking JS Timms P. Chlamydia trachomatis genital tract infections: when host immune response and the microbiome collide. Trends Microbiol. (2016) 24:750–65. 10.1016/j.tim.2016.05.007 27320172

[159] Klatt NR Cheu R Birse K Zevin AS Perner M Noël-Romas L Vaginal bacteria modify HIV tenofovir microbicide efficacy in african women. Science. (2017) 356:938–45. 10.1126/science.aai9383 28572388

[160] Cheu RK Gustin AT Lee C Schifanella L Miller CJ Ha A Impact of vaginal microbiome communities on HIV antiretroviral-based pre-exposure prophylaxis (PrEP) drug metabolism. PLoS Pathog. (2020) 16(12):e1009024. 10.1371/journal.ppat.1009024 33270801 PMC7714160

[161] Taneva E Sinclair S Mesquita PM Weinrick B Cameron SA Cheshenko N Vaginal microbiome modulates topical antiretroviral drug pharmacokinetics. JCI Insight. (2018) 3(13):e99545. 10.1172/jci.insight.99545 29997295 PMC6124523

[162] Farr Zuend C Noël-Romas L Hoger S McCorriser S Westmacott G Marrazzo J Influence of dapivirine vaginal ring use on cervicovaginal immunity and functional microbiome in adolescent girls. AIDS. (2021) 35:369–80. 10.1097/QAD.0000000000002751 33181534 PMC7924934

[163] Thurman AR Schwartz JL Ravel J Gajer P Marzinke MA Yousefieh N Vaginal microbiota and mucosal pharmacokinetics of tenofovir in healthy women using tenofovir and tenofovir/levonorgestrel vaginal rings. Plos One. (2019) 14:e0217229. 10.1371/journal.pone.0217229 31107913 PMC6527208

[164] Heffron R McClelland RS Balkus JE Celum C Cohen CR Mugo N Efficacy of oral pre-exposure prophylaxis (PrEP) for HIV among women with abnormal vaginal microbiota: a post-hoc analysis of the randomised, placebo-controlled partners PrEP study. Lancet HIV. (2017) 4:e449–56. 10.1016/S2352-3018(17)30110-8 28732773 PMC5649365

[165] Gyorke CE Kollipara A Allen J Zhang Y Ezzell JA Darville T IL-1α Is essential for oviduct pathology during genital chlamydial infection in mice. J Immunol. (2020) 205:3037–49. 10.4049/jimmunol.2000600 33087404

[166] Lavelle A Hoffmann TW Pham H-P Langella P Guédon E Sokol H. Baseline microbiota composition modulates antibiotic-mediated effects on the gut microbiota and host. Microbiome. (2019) 7:111. 10.1186/s40168-019-0725-3 31375137 PMC6676565

[167] Perelman P Johnson WE Roos C Seuánez HN Horvath JE Moreira MAM A molecular phylogeny of living primates. PLoS Genet. (2011) 7:e1001342. 10.1371/journal.pgen.1001342 21436896 PMC3060065

[168] Mestas J Hughes CCW. Of mice and not men: differences between mouse and human immunology. J Immunol. (2004) 172:2731–8. 10.4049/jimmunol.172.5.2731 14978070

[169] Bjornson-Hooper ZB Fragiadakis GK Spitzer MH Chen H Madhireddy D Hu K A comprehensive atlas of immunological differences between humans, mice, and non-human primates. Front Immunol. (2022) 13:867015. 10.3389/fimmu.2022.867015 35359965 PMC8962947

[170] Cunha GR Sinclair A Ricke WA Robboy SJ Cao M Baskin LS. Reproductive tract biology: of mice and men. Differentiation. (2019) 110:49–63. 10.1016/j.diff.2019.07.004 31622789 PMC7339118

[171] Estes JD Wong SW Brenchley JM. Nonhuman primate models of human viral infections. Nat Rev Immunol. (2018) 18:390–404. 10.1038/s41577-018-0005-7 29556017 PMC5970954

[172] Bell JD Bergin IL Schmidt K Zochowski MK Aronoff DM Patton DL. Nonhuman primate models used to study pelvic inflammatory disease caused by *Chlamydia trachomatis*. Infect Dis Obstet Gynecol. (2011) 2011:675360. 10.1155/2011/675360 21869858 PMC3160047

[173] Bakaletz LO. Developing animal models for polymicrobial diseases. Nat Rev Microbiol. (2004) 2:552–68. 10.1038/nrmicro928 15197391 PMC7097426

[174] Peña JC Ho W-Z. Non-human primate models of *Tuberculosis*. Microbiol Spectr. (2016) 4(4). 10.1128/microbiolspec.TBTB2-0007-2016 27726820

[175] Gardner MB Luciw PA. Macaque models of human infectious disease. ILAR J. (2008) 49:220–55. 10.1093/ilar.49.2.220 18323583 PMC7108592

[176] Zitsman JS Alonso-Guallart P Ovanez C Kato Y Rosen JF Weiner JI Distinctive leukocyte subpopulations according to organ type in cynomolgus macaques. Comp Med. (2016) 66:308–23. 27538862 PMC4983173

[177] Messaoudi I Estep R Robinson B Wong SW. Nonhuman primate models of human immunology. Antioxid Redox Signal. (2011) 14:261–73. 10.1089/ars.2010.3241 20524846 PMC3014769

[178] Van Esch E Cline JM Buse E Wood CE de Rijk EPCT Weinbauer GF. Summary comparison of female reproductive system in human and the cynomolgus monkey (*Macaca fascicularis*). Toxicol Pathol. (2008) 36:171S–2S. 10.1177/0192623308327415

[179] Weinbauer GF Niehoff M Niehaus M Srivastav S Fuchs A Van Esch E Physiology and endocrinology of the ovarian cycle in macaques. Toxicol Pathol. (2008) 36:7S–23S. 10.1177/0192623308327412 20852722 PMC2939751

[180] Shaikh AA Naqvi RH Shaikh SA. Concentrations of oestradiol-17beta and progesterone in the peripheral plasma of the cynomolgus monkey (*Macaca fascicularis*) in relation to the length of the menstrual cycle and its component phases. J Endocrinol. (1978) 79:1–7. 10.1677/joe.0.0790001 101640

[181] Marlin R Nugeyre M-T Tchitchek N Parenti M Hocini H Benjelloun F Modified vaccinia virus Ankara vector induces specific cellular and humoral responses in the female reproductive tract, the main HIV portal of entry. J Immunol. (2017) 199:1923–32. 10.4049/jimmunol.1700320 28760882

[182] Spear GT Gilbert D Sikaroodi M Doyle L Green L Gillevet PM Identification of rhesus macaque genital microbiota by 16S pyrosequencing shows similarities to human bacterial vaginosis: implications for use as an animal model for HIV vaginal infection. AIDS Res Hum Retrovir. (2010) 26(2):193–200. 10.1089/aid.2009.0166 20156101 PMC2835387

[183] Spear GT Kersh E Guenthner P Vishwanathan SA Gilbert D Zariffard MR Longitudinal assessment of pigtailed macaque lower genital tract microbiota by pyrosequencing reveals dissimilarity to the genital microbiota of healthy humans. AIDS Res Hum Retroviruses. (2012) 28:1244–9. 10.1089/AID.2011.0382 22264029 PMC3448102

[184] Chen Z Yeoh YK Hui M Wong PY Chan MCW Ip M Diversity of macaque microbiota compared to the human counterparts. Sci Rep. (2018) 8:15573. 10.1038/s41598-018-33950-6 30349024 PMC6197227

[185] Rhoades NS Hendrickson SM Gerken DR Martinez K Slayden OD Slifka MK Longitudinal profiling of the macaque vaginal microbiome reveals similarities to diverse human vaginal communities. mSystems. (2021) 6:e01322–20. 10.1128/mSystems.01322-20 33906914 PMC8092128

[186] Nugeyre M-T Tchitchek N Adapen C Cannou C Contreras V Benjelloun F Dynamics of vaginal and rectal microbiota over several menstrual cycles in female cynomolgus macaques. Front Cell Infect Microbiol. (2019) 9:188. 10.3389/fcimb.2019.00188 31249812 PMC6582644

[187] Hallmaier-Wacker LK Lüert S Roos C Knauf S. Lactation and menstruation shift the vaginal microbiota in captive rhesus monkeys to be more similar to the Male urethral microbiota. Sci Rep. (2019) 9:17399. 10.1038/s41598-019-53976-8 31758047 PMC6874612

[188] Mirmonsef P Gilbert D Veazey RS Wang J Kendrick SR Spear GT. A comparison of lower genital tract glycogen and lactic acid levels in women and macaques: implications for HIV and SIV susceptibility. AIDS Res Hum Retroviruses. (2012) 28:76–81. 10.1089/aid.2011.0071 21595610 PMC3251838

[189] Miller EA Beasley DE Dunn RR Archie EA. Lactobacilli dominance and vaginal pH: why is the human vaginal microbiome unique? Front Microbiol. (2016) 7:1936. 10.3389/fmicb.2016.01936 28008325 PMC5143676

[190] Yu RR Cheng AT Lagenaur LA Huang W Weiss DE Treece J A Chinese rhesus macaque (*Macaca mulatta*) model for vaginal *Lactobacillus* colonization and live microbicide development. J Med Primatol. (2009) 38:125–36. 10.1111/j.1600-0684.2008.00316.x 19367737 PMC4422182

[191] Langner CA Ortiz AM Flynn JK Kendall H Lagenaur LA Brenchley JM. The vaginal microbiome of nonhuman primates can be only transiently altered to become *Lactobacillus* dominant without reducing inflammation. Microbiol Spectr. (2021) 9:e01074–21. 10.1128/Spectrum.01074-21 34756073 PMC8579922

[192] Mahajan G Doherty E To T Sutherland A Grant J Junaid A Vaginal microbiome-host interactions modeled in a human vagina-on-a-chip. Microbiology. (2022). 10.1101/2022.03.20.485048 PMC9701078 36434666

[193] Manhanzva MT Abrahams AG Gamieldien H Froissart R Jaspan H Jaumdally SZ Inflammatory and antimicrobial properties differ between vaginal Lactobacillus isolates from South African women with non-optimal versus optimal microbiota. Sci Rep. (2020) 10(1):6196. 10.1038/s41598-020-62184-8 32277092 PMC7148372

